# An embedded computational framework of memory: The critical role of representations in veridical and false recall predictions

**DOI:** 10.3758/s13423-025-02669-7

**Published:** 2025-04-11

**Authors:** Dominic Guitard, Jean Saint-Aubin, J. Nick Reid, Randall K. Jamieson

**Affiliations:** 1 School of Psychology, Cardiff University, Tower Building, 70 Park Place, Cardiff, CF10 3AT UK; 2 Université de Moncton, Moncton, NB Canada; 3 University of Northern British Columbia, Prince George, BC Canada; 4 University of Manitoba, Winnipeg, MB Canada

**Keywords:** Serial recall, False recall, Distributional semantic models, Computational model

## Abstract

Human memory is reconstructive and thus fundamentally imperfect. One of its critical flaws is false recall—the erroneous recollection of unstudied items. Despite its significant implications, false recall poses a challenge for existing computational models of serial recall, which struggle to provide item-specific predictions. Across six experiments, each involving 100 young adults, we address this issue using the Embedded Computational Framework of Memory (eCFM) that integrates existing accounts of semantic and episodic memory. While the framework provides a comprehensive account of memory processing, its innovation lies in the inclusion of a comprehensive lexicon of word knowledge derived from distributional semantic models. By integrating a lexicon that captures orthographic, phonological, and semantic relationships within an episodic memory model, the eCFM successfully accounts for patterns of veridical serial recall (e.g., proportion correct, intralist errors, omissions) while also capturing false recall (e.g., extralist errors including both critical lures and non-critical lures). We demonstrate the model’s capabilities through simulations applied to six experiments, with lists of words (Experiments 1A, 1B, 2A, and 2B) and non-words (Experiments 3A and 3B) that are either related or unrelated semantically (Experiments 1A and 1B), phonologically (Experiments 2A and 2B), or orthographically (Experiments 3A and 3B). This approach fills a computational gap in modelling serial recall and underscores the importance of integrating traditionally separate areas of semantic and episodic memory to provide more precise predictions and holistic memory models.

Verbal memory plays a pivotal role in all facets of our lives, yet it is fundamentally imperfect and prone to errors (Bartlett, 1932). Understanding these errors is one of the most important and challenging questions in cognitive science, with significant theoretical and practical ramifications (Henson, 1998). Among the various memory errors, false recall or extralist errors, which occur when you recall an unpresented word like *'club*' after studying a list of words like *'golf, member, ball, dance, organization, house*' (Tehan, 2010), poses a significant challenge for short-term memory models. In this study, we take up the challenge and propose a mechanistic explanation of false recall based on our recently proposed Embedded Computational Framework of Memory (eCFM: Guitard et al., 2025).

False recall (Deese, 1959; Roediger & McDermott, 1995) has been instrumental in revealing the reconstructive nature of human memory, with substantial implications for our understanding of memory processes (see Chang & Brainerd, 2021). Despite its importance, most empirical and computational efforts have focused on recognition tasks (e.g., Reid & Jamieson, 2023; Arndt & Hirshman, 1998), where participants identify whether a word was studied or is new after studying a list, and free recall tasks (e.g., Kimball et al., 2007; Sirotin et al., 2005), where participants recall all words they remember from a list without considering their order. These paradigms have yielded a rich and consistent pattern of results across semantically related (e.g., Deese, 1959; Roediger & McDermott, 1995), phonologically related (e.g., Sommers & Lewis, 1999), and orthographically related words (e.g., Ballardini et al., 2008) and non-words (e.g., Zeelenberg et al., 2005). Taken together, those results demonstrate the generality of the phenomenon over the three dominant principal linguistic dimensions.

While prior research in recognition and free recall has provided valuable theoretical and practical insights (e.g., Benedek & Schetky, 1987; Loftus, 1996, 2003; Schacter et al., 1997), serial recall offers a unique opportunity to explore a rich and precise pattern of veridical and erroneous memories unmatched by other memory protocols (Henson, 1998). Here, we leverage this unique opportunity to investigate false recall through both empirical and computational methods. This approach seeks to address the difficulties that traditional memory models (e.g., Brown et al., 2007; Henson, 1998; Murdock, 1993; Nairne, 1988, 1990; Saint-Aubin et al., 2021) have faced in accounting for memory errors at word-level precision. By systematically examining these errors with semantically, phonologically, and orthographically related and unrelated memoranda, we aim to provide a mechanistic account of false recall that overcomes previous practical limitations in modeling word-level recall and offers a rich empirical database for model development.

In serial recall tasks, participants study a list of words and then are tasked with recalling them in order. In addition to correct recall, participants can make various errors, such as omissions (failing to recall a word), intralist errors (recalling a word in a different position), and false recall (i.e., recalling a word that was never presented; often called extralist errors or intrusions). These errors have been observed across the lifespan and have significantly influenced the development of computational models and the field’s understanding of human memory (e.g., Henson, 1998; Maylor et al., 1999; McCormack et al., 2000; Tehan, 2010). They can occur in lists artificially created using the DRM paradigm (Deese, 1959; Roediger & McDermott, 1995) in which studied materials (e.g., *bird, peace, white, beak, bar*) are related to a specific critical lure (e.g., *dove*) (Tehan, 2010) or in lists without such structure (e.g., Maylor et al., 1999; McCormack et al., 2000). Therefore, a comprehensive account should be able to precisely capture both instances, which was our goal in this study.

While most memory models can account for the differences in the rates at which the different categories of error occur (e.g., Henson, 1998; Maylor et al., 1999), they fall short in making word specific predictions. For example, models might predict the probability of false recall (e.g., predicting the total number of errors or the distribution of errors) but they do not actually recall words, making it difficult to predict memory errors in a manner directly comparable to people’s recall behavior. In other words, most memory models neither account for the rich lexical-semantic relationships of the studied materials nor produce behavior that can be directly evaluated against memory performance.

This is an important disconnect with empirical investigations, given the growing evidence that specific verbal characteristics of the to-be-remembered information can have drastic consequences on memory performance (see e.g., Guitard et al., 2018). Indeed, it is now well-established that various lexical (e.g., orthographic, phonological) and semantic properties affect serial recall performance (e.g., Guitard et al., 2018; Hulme et al., 1991, 2003; Majerus, 2019; Neath et al., 2022; Roodenrys et al., 2022; Oberauer et al., 2018). Therefore, building models without the ability to account for these important relationships is likely to fall short in understanding the complex influence of our linguistic knowledge on verbal memory performance.

Among these errors, false recall poses the most significant challenge because it requires a lexicon or long-term memory that captures the richness of lexical representation, that traditional models lack. Consequently, current short-term memory models either fail to produce false recall or struggle to do so with precision. To address this, models need to capture the influence of complex lexico-semantic knowledge, which requires accurately representing the relationships between studied words. Traditionally, many serial recall models have been agnostic about these relationships by using randomly generated vectors (i.e., arbitrary sequences of numbers without any inherent meaning) to represent item information (e.g., Murdock, 1993; Brown et al., 2007; Henson, 1998; Nairne, 1988; Saint-Aubin et al., 2021; cf. Raaijmakers & Shiffrin, 1981). While this approach allows researchers to focus on the structural components of model architecture and has provided valuable theoretical insights (e.g., Osth et al., 2020), it remains atheoretical regarding the relationship between information in the studied lists and information from our past experiences. As a result, models with randomly generated representations often fail to capture the full picture of lexical relationships formed by language experience, thereby missing the complexity and structural richness inherent in natural language (e.g., Johns & Jones, 2010).

To overcome the full complexity of false recall and human memory in general, traditional memory models (e.g., Brown et al., 2000, 2007; Burgess & Hitch, 1999; Henson, 1998; Nairne, 1990; Murdock, 1995) need a solution. Here, we propose a solution that has shown initial success in accounting for false memory in recognition (e.g., Johns et al., 2012, 2020; Osth et al., 2020) and free recall (Kimball et al., 2007; Sirotin et al., 2005): structured word representations that reflect the nuanced interrelations between words as they are known to individuals.

Traditionally, like serial recall models, recognition and free recall models have utilized randomly generated vectors to represent memory information (e.g., Arndt & Hirshman, 1998; Hintzman, 1984, 1986, 1988; Raaijmakers & Shiffrin, 1981; Shiffrin & Steyvers, 1997). However, recently, researchers have highlighted the potential of integrating distributional semantic models (DSMs), like Latent Semantic Analysis (LSA; Landauer & Dumais, 1997), BEAGLE (Jones & Mewhort, 2007), and the Random Permutation Model (Sahlgren et al., 2008; Recchia et al., 2015) into established memory models to capture the complexity of word knowledge relative to language experience. DSMs are computational models that represent the meanings of words based on their distributional properties in large text corpora. They provide a way to understand complex relationships between words by analyzing patterns of co-occurrence, thereby creating vector representations of word meanings that captured nuanced relationships between words both to one another and, more critically, to all words stored in the lexicon (Reid & Katz, 2018; Lenci, 2018).

Memory models that have integrated DSM-based lexical representations into established recognition and free recall models have significantly advanced our understanding of memory. For example, the Recognition through Semantic Synchronization (RSS) model by Johns et al. (2012, 2020; see also Chang et al., 2025) incorporates DSM representations and accounts for numerous false recognition phenomena observed in DRM paradigms with an insightful level of precision. This includes increased false recognition when more associates are studied (Robinson & Roediger, 1997), situational knowledge-based false recognition (Cann et al., 2011), and false recognition at the individual item level (Gallo & Roediger, 2002; Roediger et al., 2001; Stadler et al., 1999).

Likewise, Osth et al. (2020) used BEAGLE's semantic representations alongside the diffusion decision model (Ratcliff, 1978) to investigate global similarity effects on recognition memory performance. They found that global semantic similarity, as measured by BEAGLE, impaired recognition performance for both targets and lures, with stronger impairments for lures.

More similar to our current approach, Reid and Jamieson (2023) employed DSM representations derived from LSA within the MINERVA 2 framework to simulate false recognition for words, sentences, and even metaphors. The combined model was called “MINERVA S”, with the S standing for “semantics” (Reid & Jamieson, 2022). With the LSA semantic representations, the model was able to account for false recognition of critical lures from DRM word lists (Arndt & Hirshman, 1998; Roediger & McDermott, 1995), for literal sentences containing similar ideas (Bransford & Franks, 1971), and for figurative expressions containing similar metaphorical themes expressed in different words (Reid & Katz, 2018, 2022; Yang et al., 2022). The model was recently extended to account for orthographic, phonological and semantic information in an extension called MINERVA OPS (Reid et al., 2023a, b) and was able to account for the effect of false recognition across orthographic, phonological, and semantic materials (see also Chang & Johns; 2023; Chang et al., 2025; Cox et al., 2011; Osth & Zhang, 2023; Steyvers, 2000; Zhang & Osth, 2024). In this study, we build on these insights to extend the methods to the problem of short-term memory.

Beyond recognition, DSMs have shown their versatility and potential in explaining the complexities of human memory in other contexts (Gatti et al., 2022; Jamieson et al., 2022; Johns & Jones, 2010; Johns & Jamieson, 2019; Jones, 2019; Kelly et al., 2020; Kimball et al., 2007; Mewhort et al., 2018; Morton & Polyn, 2016; Petilli et al., 2024; Polyn et al., 2009). For example, Sirotin et al. (2005) and Kimball et al. (2007) integrated similarity scores based on LSA (Landauer & Dumais, 1997) and word association space (WAS; Steyvers et al., 2005) into Raaijmakers and Shiffrin’s Search of Associative Memory (SAM) model (1980, 1981). This approach effectively explained patterns of false recall and memory errors using a relatively small lexicon (e.g., 750 words; 250 words). Mewhort et al. (2018) employed a large lexicon containing 39,076 words represented by BEAGLE vectors within a holographic model for recall. This model successfully accounted for phenomena such as the Hebb effect (memory improvement for repeated sequences), the von Restorff effect (enhanced memory performance for a distinctive item), and the release of proactive interference (improved memory performance following the introduction of a novel semantic category after several trials of words from the same category). Because the models are tested directly against the same word lists used in experiments, the demonstrations take modelling of human memory from demonstrations in principle (with random vectors) to demonstrations in particular (with word specific DSM word vectors).

Inspired by these advancements, we have moved beyond traditional conceptions of short-term memory models to investigate the opportunities gained by embedding semantic information in memory for studied lists (Guitard et al., 2025). By integrating successful aspects of episodic and semantic memory models from recognition (e.g., Reid & Jamieson, 2022) and recall (e.g., Mewhort et al., 2018), we developed the Embedded Computational Framework of Memory (eCFM) to illustrate how embedding a lexicon in a model of episodic memory can enhance the predictive specificity of short-term memory models.

The eCFM is a computational model that incorporates structured word representations, encoding, storage, retrieval, and decision processes. By embedding semantic structures such as those derived from Latent Semantic Analysis (LSA; Landauer & Dumais, 1997) into the episodic memory framework of MINERVA 2 (Hintzman, 1986), the eCFM achieves a more nuanced and accurate prediction of verbal memory performance. This approach aligns with the principles championed by Murdock and Lewandowsky through TODAM and its iterations (e.g., Murdock, 1982, 1993, 1995, 1997, 2006; Lewandowsky & Murdock, 1989), which demonstrated the utility of episodic memory models in capturing short-term memory processes.

This integration follows a well-established tradition in cognitive science, emphasizing that short-term memory processes are not isolated but emerge from broader memory processes (e.g., Oberauer, 2009; Cowan, 1988, 2019, Cowan et al., 2024; Murdock, 1995). While there has been a long-standing and vigorous debate regarding the division between episodic and short-term memory systems (see Baddeley, 2012; Morey, 2018; Murdock & Kahana, 1993, Nairne, 1990; Shallice & Warrington, 1970; Surprenant, & Neath, 2009), this discussion falls outside the scope of the current work. Regardless of one’s preferred theoretical perspective, the key solution proposed in this study—embedding a lexicon into a memory model—offers a robust and practical approach to addressing limitations in short-term memory models while remaining compatible across different theoretical frameworks.

By demonstrating how semantic and episodic processes interact, eCFM offers insights into the mechanisms underpinning verbal memory, paving the way for more comprehensive and integrated approaches to memory modeling. For example, in our recent demonstrations with the eCFM model, we have shown its capability to capture both item-specific and overall predictions of various phenomena, such as the beneficial effect of semantic relatedness in serial recall and its reduction in serial reconstruction, the influence of semantic relatedness on migration errors, the interaction between task difficulty and semantic relatedness, the detrimental effects of semantic relatedness on order information, and the influence of the number of studied words related to the critical lure on the likelihood that participants will falsely recall semantic associates (Guitard et al., 2025). The eCFM appears well-suited for assessing the value of embedding a lexicon to capture patterns in word specific false recall across materials. However, our original implementation that considers only semantic information is incomplete because other lexical characteristics such as orthographic and phonological information, both of which affect verbal short-term memory performance, were unrepresented and thus unconsidered (e.g., Cowan et al., 2022; Guitard et al., 2018; Roodenrys et al., 2022; Saint-Aubin et al., 2023). To address the shortcoming we have expanded our model’s lexicon to include the orthographic and phonological relationships between words, in addition to their semantic relationships.

To gain traction on the issue, we demonstrate how the eCFM lexicon can be extended to capture orthographic and phonological relationships, building on the recent work of Reid et al. (2023a, b). We systematically investigated the model's ability to capture false recall across six experiments, encompassing semantic (Experiments 1A and 1B), phonological (Experiments 2A and 2B), and orthographic (Experiments 3A and 3B) information. Each experiment tests the model’s ability to both handle lists of related and unrelated words using traditional critical lures (e.g., Tehan, 2010) and move beyond this metric to capture common extralist errors that might have served as critical lures but have not been traditionally classified as critical lures in experimental work (e.g., Maylor et al., 1999; McCormack et al., 2000). Additionally, we evaluated whether a more comprehensive lexicon, which simultaneously captures orthographic, phonological, and semantic relationships amongst words, can more accurately account for the specificity of these memory errors.

In summary, the aim of this study was to evaluate whether embedding a lexicon of structured representations that capture word relationships can overcome the current limitations of immediate ordered recall models, enabling them to predict human memory errors at an improved level of precision for implementation in existing models of episodic memory.

## Embedded computational framework of memory

As mentioned, the eCFM is a computational model that incorporates a lexicon along with encoding, storage, retrieval, and decision processes (Guitard et al., 2025). The original model integrated structured word representations from the LSA model of semantic memory (Landauer & Dumais, 1997) into the MINERVA 2 (Hintzman, 1986) model of episodic memory. In this study, we have extended the model’s lexicon and applied the eCFM to serial recall tasks involving semantically, phonologically, and orthographically related lists (Experiment 1A, Experiment 2A, Experiment 3A) as well as unrelated lists (Experiment 1B, Experiment 2B, Experiment 3B) of words and non-words. The following sections will briefly describe the model’s architecture and representations, after which we will apply the model to a series of experimental tests (for an illustration of each parameter see Appendix D).

### Item representation

To model false remembering in serial recall, the eCFM involves the memorial representation of the study list as well as a complete lexicon where words are represented based on their similarity. In our current model version, we use semantic, phonological, and orthographic lexical representations that match the task design. This approach aligns with recent empirical and computational advances suggesting that list structure can focus encoding on relevant lexico-semantic dimensions (e.g., Caplan, 2023; Caplan & Guitard, 2024a, b). For example, participants presented with a list of related words might process those words for meaning, whereas participants presented with a list of nonsense letter strings might process those items for orthography. In the final demonstrations, after showing the model's ability to track false recall with subsetted representations, we illustrate how it can operate upon integrated representations (that include orthographic, phonological, and semantic information) to capture false recall for both phonological and semantic information.

#### Semantic representations

In the eCFM, semantic representations are derived using LSA, a widely-used DSM (Landauer & Dumais, 1997). To derive these vectors, we constructed a word-by-document matrix from the Touchstone Applied Science Associates Inc. (TASA) corpus, performed singular value decomposition of that matrix, and represented each word’s meaning as a reduced 300-dimensional projection. The LSA vectors are available on the OSF page associated with this project. For our simulation, we applied several constraints to refine the lexicon to better reflect participants' language experience. First, we limited the vectors to include only words from the SUBTLEXus database (Brysbaert et al., 2012) with a Zipf word frequency between 1 and 7. Second, we included only specific parts of speech that typically reflect studied and extralist errors produced by participants: adjectives, adverbs, names, nouns, numbers, verbs, and interjections. Additionally, we removed a list of 442 problematic words due to their high co-occurrence with all words in the lexicon (e.g., "all," "and," "a"), as detailed on the **OSF** page associated with this manuscript.

Furthermore, we included only words for which we can derive orthographic, phonological, and semantic representations. This ensured a consistent lexicon size across simulations and mitigated the risk of differences being attributed to variations in lexicon size depending on which lexical characteristic we were examining. Consequently, the final lexicon comprised 41,005 words. We believe this refined lexicon reasonably represents the vocabulary of a typical participant in our empirical study. These semantic vectors were employed to represent words in Experiment 1, where we manipulated semantic similarity.

#### Phonological Representations

To capture phonological representations, we employed a recently proposed method by Parrish (2017) that breaks the phonemes of a word down into their sound features. Because phonemes are not pronounced discretely but depend on the phonemes that come before and after (i.e., coarticulation), the model uses an “interleaved bigram” scheme where the sound features of adjacent phonemes interact. For instance, for the word “knee”, the sound features of the phoneme /n/, “alveolar” and “nasal”, interact with the sound feature of /i/, “front”, “high”, “unrounded”, “vowel”, to produce the following eight pairs of sound features: alveolar-front, alveolar-high, alveolar-unrounded, alveolar-vowel, nasal-front, nasal-high, nasal-unrounded, nasal-vowel. For each word, these pairs are stored in a word-by-sound matrix. We then performed singular value decomposition on that matrix and represented each word as a 300-dimension projection to maintain consistency with the dimensionality of our semantic representations. Reid et al. (2023a, b) previously employed these vector representations within MINERVA OPS to capture false recognition effects for study lists made of up of phonological associates. Like with the semantic vectors, we applied the same constraints to subset the lexicon. The final lexicon was composed of 41,005 words. These phonological vectors are accessible on the **OSF** page associated with this project. We used these vectors to represent memory for words in Experiment 2, where we manipulated phonological similarity.

#### Orthographic representation

To capture orthographic representations for non-words, we created vector representations inspired by the open-bigram scheme from SERIOL and SERIOL2 (Whitney, 2001; Whitney & Marton, 2013). In that model, words are encoded as non-contiguous bigrams with specific activation rules. The activation weights were assigned based on the number of intervening letters, employing values of 1, 0.7, and 0.5 for bigrams with 0, 1, and 2 intervening letters (see Hannagan et al., 2011). Special markers denoted by * were used to represent the beginning and end of words and were treated like extra letters in the word, which helps to emphasize letters on the edge of the word. As an example, for the word “cat”, the bigrams *c, ca, at, t* would have activation values of 1, the bigrams *a, ct, and a* would have activation values of 0.7, and the bigrams *t and c* would have activation values of 0.5. These bigrams were recorded in a word-by-bigram matrix, with weights added rather than counts. For words with repeated bigrams, the weights were summed. The matrix was then reduced to 300 dimensions using singular value decomposition, maintaining consistency with the dimensionality of our semantic and phonological representations already described. This same representational technique was used by Reid et al. (2023a, b) in combination with MINERVA S to model false recognition of pseudowords in item-method directed forgetting. The lexicon was composed of all possible three-letter combinations (e.g., 17,576 three-letters combinations) and is accessible on the **OSF** page associated with this project. The orthographic representations were used to represent items in Experiment 3, where orthographic similarity was manipulated for three-letter non-words. The same technique was also used to derive orthographic representations for the 41,005 words used in the simulation with the full representations (orthographic, phonological, semantic) in the final demonstrations.

### Order representation

In the eCFM, we assume participants encode words at their studied serial positions and then use these serial positions as cues for recalling words at test. This addition is an important enhancement to the traditional model of episodic memory MINERVA 2 (Hintzman, 1986), and is necessary to capture serial recall performance. Serial position representations are based on item-independent context models (e.g., see Logan & Cox, 2023; Osth & Hurlstone, 2023). More exactly, to represent order information, we generate a random vector of dimensionality *n* for the first position where each dimension takes the value of a random deviate from a Normal distribution with mean 0 and standard deviation $1/\sqrt{n}$ (e.g., Jones & Mewhort, 2007; Murdock, 1982). Subsequently, for each successive serial position, a new vector is generated by copying the representation from the preceding serial position and sampling a new deviate from the same normal distribution for each dimension with probability *d* that controls the degree of similarity over successive serial position representations. This approach creates a series of vector-based serial position representations that vary in similarity as a function of serial distance. As will become clear when we describe retrieval more fully, the model suffers the most retrieval interference from events encoded at immediately adjacent serial positions and the least retrieval interference from events at the most distant serial positions, with the value of *d* controlling the degree of interference.

### Encoding

We posit that individuals encode a study list as a sequence of traces, where each trace contains both the relevant serial position representation, and the word presented at that serial position. To translate this assumption into computational terms, we utilize a matrix, **M**. Each row in this memory matrix constitutes a 600-dimensional vector. The first 300 dimensions encode serial order information (serial position) and the second 300 dimensions encode item information (lexical representation). Therefore, for a study list consisting of six items, **M** is a 6 x 600 matrix.

We assume that memory encoding for a studied word and its corresponding order information is imperfect. In the current implementation, items presented earlier are better encoded, and the last item is encoded equally well as the previous one. This assumption aligns with the idea that people have more opportunities to use maintenance strategies, such as rehearsal (e.g., Bhatarah et al., 2009; Rundus, 1971), that attentional resources deplete as a function of serial position (e.g., Popov & Reder, 2020), and models suggesting an activation gradient (e.g., Page & Norris, 1998). Additionally, this is consistent with previous models indicating that later items are less likely to suffer from retroactive interference (e.g., Nairne, 1990; Saint-Aubin et al., 2021) or are protected because of their privileged position at the end of the list (e.g., Henson, 1998; Brown et al., 2007). Although other encoding assumptions are possible, our goal is to demonstrate the value of embedding a lexicon to capture specificity, so we have adopted general assumptions rather than committing to a specific theoretical framework. This serves as a proof of concept for the value of embedding a lexicon, and further work is ongoing to improve these encoding mechanisms.

To incorporate our encoding assumptions into the eCFM, we copy each feature in a trace at serial position *p* with probability *L*_*p*_,

1 $${L}_{p}=\left\{\begin{array}{cc}L-\left(\text{p}-1\right)\text{g}& , p<LL\\ L-\left(\text{p}-2\right)\text{g}& , p=LL\end{array}\right.$$

Here, *L* corresponds to the effectiveness of encoding the first presented item in a study list, *p* represents the serial position, *g* signifies the rate at which encoding diminishes with serial position, and *LL* denotes the total number of items in the studied list (i.e., the list length). As shown in Eq. 1, each item is encoded less effectively than its predecessor at a rate *g*, with the exception of the last item that is encoded as effectively as the second-last item (for an illustration of each parameter and its influence on encoding see Appendix D).

### Retrieval

In the model, retrieval is parallel, cue-specific, and similarity driven. This means that when a cue is introduced (i.e., the intact serial position representation), it triggers the retrieval of memory traces that are similar to it, including those from all adjacent serial positions; albeit to differing degrees controlled by *d*. Crucially, because a cue retrieves whole memory traces, and these traces contain both serial position (order) and word (item) information, a cue that includes only serial position information retrieves the associated word information it co-occurred with at study.

More specifically, after encoding information into memory, an intact cue (e.g., representing the first serial position to recall the first word) is presented during recall. This cue interacts with all serial position representations in memory in parallel. The decision process arises from this reconstruction. Due to the similarity-driven nature of retrieval, the cue activates the most similar traces most strongly. The retrieved trace, or *echo*, is then used to extract the word information associated with that representation. A decision to recall a specific word is made based on the cosine similarity between that item information in the echo and all item representations in the lexicon. If the cosine similarity between the echo and the item it is most similar to in the lexicon is greater than a recall threshold, the item is reported (see also, Johns et al., 2020). In the next section, we describe in more detail how this process is applied to account for serial recall performance.

### Serial recall simulation

In the eCFM, serial recall unfolds over two computational steps. First, after the presentation of items, recall at each serial position *LL* is simulated by presenting the relevant serial position representation as a cue, **q**, and retrieving the corresponding echo, **e**, from memory,

2 $$\mathbf{e}=\sum\nolimits_{i=1}^{i=m}{\left(\frac{\sum_{j=1}^{j=n/2}{q}_{j}\times {M}_{ij}}{\sqrt{\sum_{j=1}^{j=n/2}{q}_{j}^{2}}\sqrt{\sum_{j=1}^{j=n/2}{M}_{ij}^{2}}}\right)}^{3}\times {M}_{i\bullet }$$

where *q*_*j*_ is feature *j* in the cue, *M*_*ij*_ is feature *j* in trace *i* in memory, 1…*n/2* is the dimensionality of the serial position cue in each memory trace (i.e., dimensions 1…300), and *m* is the number of traces in memory (i.e., the length of the study list, or *LL*, in the simulations that follow).

In psycholinguistic terms, the echo corresponds to a mental representation, a lemma, encapsulating the underlying thought behind a language expression. Usually, this echo, **e**, mirrors the word at the cued serial position. However, due to the fact that related study words have similar representations and that words in the embedded lexicon have incidental similarities to one another, retrieved information from memory holds the potential to produce a false recall (i.e., an extralist error).

Secondly, we compute the similarity between the information retrieved in the last 300 dimensions of the echo (i.e., dimensions 301…600), representing the lexical features of the item, and every word within the lexicon. If the word in the lexicon with the highest cosine similarity surpasses a specified recall threshold, *T*, it is chosen for report. Conversely, if no word in the lexicon meets or exceeds *T*, no word is reported, resulting in an omission. To prevent continuous retrieval of the same item (repetition error) after it has been recalled, the model can suppress report of an already reported word at rate *s*. Importantly, this does not stop the model from recalling a word multiple times within a single trial, but rather indicates some level of resistance to such repetition (see e.g., Armstrong & Mewhort, 1995; Cowan & Hardman, 2021; Greene, 1990).

Finally, we assess the model’s recall using scoring methods commonly applied to human recall performance in serial recall studies. For example, for proportion correct, a point is awarded only if the item reported at serial position *p* matches the item presented at that position. An omission occurs when no item is reported at serial position *p* because the level of similarity between the echo and the items in the lexicon was below the recall threshold, *T*. An intralist error, also known as an order error, occurs when the item reported at position *p* was presented at a different serial position in the study list. An extralist error, also known as a false recall, occurs when the extralist error matches a targeted unstudied word. This happens when the model recalls a word that was not part of the list: an extralist error can be scored as a critical lure if the reported word at serial position *p* corresponds to the critical lure; if not, it is scored as a general extralist error.

In each experiment, we simulated recall for 100 simulated participants within each condition and report average performance over those 100 simulated participants. This approach enables us to present model results in the same way as empirical results: as average serial position functions plus proportions of errors and false recalls across different scoring procedures.

In summary, the eCFM assumes that individuals encode words-in-position at study to varying degrees of accuracy. During recall, they use serial positions as cues to retrieve echoes that include the item information. The item information in the echo is compared to all words in the lexicon, and the best matching word is reported. The reported words are then compared to the studied list and scored similarly to participant behavior in terms of correct recalls, order errors, and false recalls. We report results from the model in the same way as we report results from participants in corresponding experiments using the same word lists and test conditions.

### Serial recall demonstration

Before presenting the empirical and computational demonstrations, we first ensure readers' understanding of the model by presenting simulations of serial recall with three lists that manipulate the number of related words. Specifically, we conducted simulations with 6 related words (RRRRRR), 3 related words and 3 unrelated words (RRRUUU), and 6 unrelated words (UUUUUU) to the critical non-presented word “bass.”

**Fig. 1:**
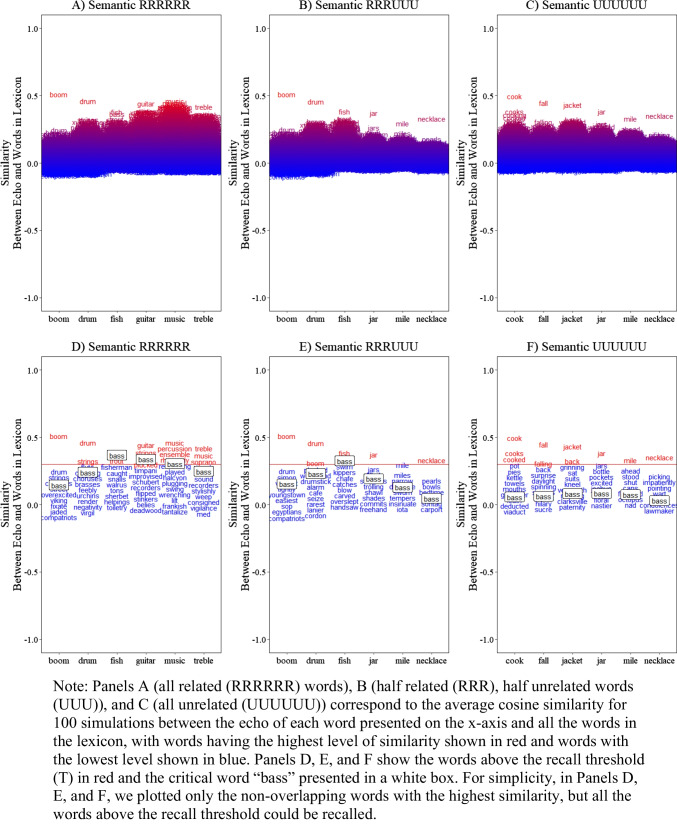
Illustration of the model simulation for serial recall as a function of the number of related words associated with the critical lure “bass” (six related, three related, none related)

A total of 100 simulations was conducted, for each list with the following parameters: *L* = 0.265, *g* = 0.03, *d* = 0.3, *T* = 0.30, *s* = 0, and the semantic representations (see Table 1). The results are presented in Fig. 1. In Panels A, B, and C, you can see the echo similarity retrieved for each position with all the words in the lexicon. The red region corresponds to the words with the highest level of similarity/activation, echoing the activated long-term memory concept of Cowan (1988, 2019; Cowan et al., 2024). In Panels D, E, and F, for simplicity, we removed the overlapping words and show the similarity to the critical word “bass” for each position and each list, along with the recall threshold (the red line). We also highlight the words above the recall threshold in red, indicating words that could be recalled. Words below the line would not be recalled and would result in an omission if no words are above the recall threshold.

**Table 1:** Parameters and vectors used for each demonstration

Demonstration	Study	Materials	Vectors	L	d	g	T	s
0	Model Illustration	Semantic Words	Semantic	0.265	0.30	0.03	0.30	0
0	Model Illustration	Semantic Words	Random	0.265	0.30	0.03	0.30	0
1	Experiments 1A and 1B	Semantic Words	Semantic	0.265	0.30	0.03	0.30	0
2	Experiments 2A and 2B	Phonological Words	Phonological	0.215	0.30	0.03	0.30	0
3	Experiments 3A and 3B	Orthographic Non-words	Orthographic	0.190	0.30	0.03	0.30	0
4	Experiments 1A and 1B	Semantic Words	Orthographic, Phonological, Semantic	0.250	0.30	0.03	0.40	0
5	Experiments 2A and 2B	Phonological Words	Orthographic, Phonological, Semantic	0.215	0.30	0.03	0.40	0

As shown, the likelihood of recalling the critical word “bass” increased with the number of related words. The word “bass” shows the highest similarity when all of the studied words are related, relative to when half of or none of the studied words are related. This demonstration captures a critical assumption in false recall: when the number of studied words associated with the critical word increases, the likelihood of falsely recalling that critical word also increases (see, e.g., Spens & Burgess, 2024; Robinson & Roediger, 1997; Guitard et al., 2025). It also shows that recall in the model is not limited to the words in the study list or to some subset of critical lures to measure false recall; rather, the model predicts recall of every word in the lexicon and presents a picture of memory that extends to the whole lexicon that a participant possesses when they arrive to a laboratory experiment.

**Fig. 2:**
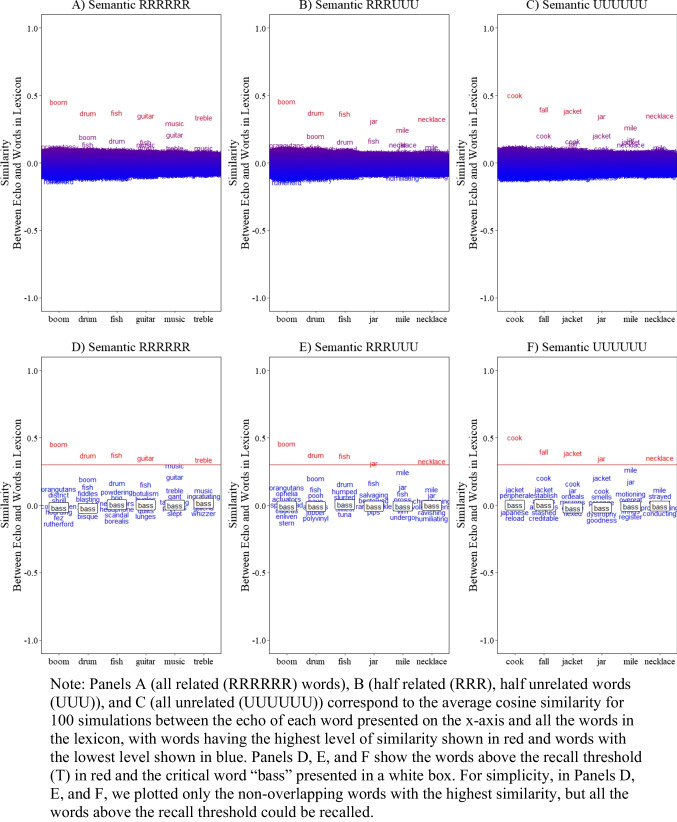
Illustration of the model simulation for serial recall as a function of the number of related words associated with the critical lure “bass” (six related, three related, none related) with *orthogonal representations* (random vectors)

To help readers understand the value of embedding structured word representations, we re-ran the simulations in Fig. 2, but this time we replaced the structured semantic representation with random and thus approximately orthogonal representations, as is traditionally used in memory models. Specifically, we created representations for each word in the lexicon sampling values from a normal distribution with a mean of 0 and a standard deviation of $1/\sqrt{n}$ (e.g., Jones & Mewhort, 2007; Murdock, 1982).

The results show that the model can still recall the presented words, as this is primarily driven by encoding and retrieval of studied words. However, there are two notable distinctions from these simulations. First, the level of similarity/activation is much lower for all non-presented words because they are approximately orthogonal and thus do not rise to the level of potential extralist errors. Second, the critical word now has a similarity level near 0 and is unaffected by the number of related words in the studied list, clearly demonstrating the value of having structured representations to make meaningful predictions beyond the typical distinction between correct versus incorrect recall.

Additionally, we observe that traditional veridical memory performance, while capable of capturing memory for specific words, makes little theoretical sense. It suggests that only the presented words would be activated, which violates key underlying assumptions of memory models (e.g., Cowan et al., 2024). This highlights the importance of using structured representations in making more comprehensive, item specific, and theoretically sound predictions.

With these demonstrations in mind, we now proceed to an examination of whether we can empirically (and computationally) capture false recall performance for lists of words that are related or unrelated semantically (Experiments 1A and 1B), phonologically (Experiments 2A and 2B), and orthographically (Experiments 3A and 3B).

## Semantic: Experiment 1A & Experiment 1B

In this experiment, we investigated the influence of semantic information on the production of false memories. To do so, we tested memory for semantically related (Experiment 1A) and semantically unrelated lists (Experiment 1B) in serial recall. Our goal across these two experiments was to provide experimental data under a common experimental protocol to evaluate if the eCFM, by embedding a lexicon into a simple memory model, can capture false recall. We present Experiments 1A and 1B together to facilitate understanding of the key empirical and simulation findings.

### Method

#### Participants

The experiment followed the prior demonstration by Tehan (2010) in which 40 participants were recruited, and false memories were detected in short-term immediate and delayed serial recall tasks. However, to ensure stability in estimation for computational modeling, we increased the number of participants to 100 in each experiment. A one-sample *t*-test sensitivity analysis conducted with G*Power 3.1.9.7 (Faul et al., 2007) with alpha set to 0.05 and power set to 0.95 revealed that 100 participants would allow us to detect a small effect of Cohen’s *d* = 0.33. For all experiments, we adopted this sample size to ensure reliable estimation.

One hundred participants recruited via Prolific (https://www.prolific.com/) took part in Experiment 1A and 100 were recruited to take part in Experiment 1B. All participants received £9.00 per hour (pro-rated) for their participation. To participate in this experiment, participants had to meet the following criteria: (a) be a native speaker of English, (b) have nationality from the United Kingdom, United States, or Canada, (c) have normal or corrected-to-normal vision, (d) have no cognitive impairment or dementia, (e) have no language-related disorders or literacy difficulties, (f) be between 18 and 25 years of age, and (g) have an approval rating of at least 90% on prior submissions at Prolific. All inclusion criteria were self-reported by the participants except for the approval rating, which is computed by Prolific. In addition, a new sample of 100 participants was recruited for Experiment 1B.

In Experiment 1A, the participants had a mean age of 22.09 years (SD = 2.05). Among the 100 participants, 60 self-identified as female, 36 as male, and 4 preferred not to specify their gender. In Experiment 1B, the participants had a mean age of 22.90 years (SD = 1.81). Of the 100 participants, 64 self-identified as female, 32 as male, and 4 preferred not to specify their gender.

#### Materials

The stimuli in Experiment 1A included 20 word lists, each comprising six words that were thematically related to an unpresented critical lure. These semantic related lists were created using the University of South Florida’s free association, rhyme, and word fragments norms (Nelson et al., 2004). Specifically, 20 target one-syllable words, ranging from 3 to 5 letters, and their stronger associates were selected. These words were selected to maximize the likelihood of detecting a false recall for the critical unstudied but related lures. In Experiment 1B the same words were used but the lists were re-arranged to minimize the similarity among words within presented lists. The stimuli for both experiments are presented in Appendix A. For both stimulus sets, we presented the mean cosine similarity among the words and the critical lure for each list. The average cosine similarity across all lists for Experiment 1A was higher (*M* = .412, *SD* = .102) than the average cosine similarity for Experiment 1B (*M* = .221, *SD* = .032) as revealed by a Bayesian *t*-test with a Bayes factor (BF10) greater than 10,000.

In both experiments, all participants were tested on all 20 lists which were presented in randomized order for each participant, but the order of the words within a list was fixed. Therefore, words within each list were always presented in the same position, but the lists themselves could be presented in a different order.

#### Ethics

All experiments were approved by the School of Psychology Ethics Committee of Cardiff University.

#### Procedure

All the experiments were programmed using PsyToolKit (Stoet, 2010, 2017), and participants took approximately 12 minutes to complete the experiment. The experiment proceeded at the participant's own pace; they initiated each trial by pressing the space bar within 60 seconds after completing the preceding trial. If the participant did not initiate the next trial within the 60-second window, the next trial was automatically presented to ensure the procedure was completed within the expected timeframe.

During the trials, the six words to be remembered were presented sequentially on the computer screen at a rate of one word per second (1000 ms on, 0 ms off), with the words displayed in white lowercase 30-point Times New Roman font against a black background at the center of the screen. After the presentation of the last word, participants engaged in a parity judgment task lasting 6 seconds. In this task, a random integer from 0 to 9 appeared at the center of the screen, with the instruction “Press the Z key for odd number” displayed at the bottom left and “Press the M key for even number” at the bottom right. During these 6 seconds, participants were instructed to complete as many parity judgments as possible. The parity judgments were not analyzed in the present study. However, they are available on the **OSF** page associated with this manuscript. The parity judgment task was included to increase the likelihood of detecting specific false recalls, based on the higher number of false recalls in previous demonstrations of performance under a delayed recall compared to immediate recall protocol, as shown by Tehan (2010). However, based on our recent work in immediate serial recall (e.g., Guitard et al., 2025) without such delays, we anticipate that the results will be similar.

Immediately after the parity judgment task, a recall cue (“Type the first word”) appeared at the top of the computer screen. Participants were required to type the words in the order they were presented, pressing the return key after typing each word. Once a response was entered, the typed word was cleared from the screen, and the instruction was updated to “Type the second word.” This process continued until all responses were entered. Participants were not allowed to go back and change a response once it was registered.

The procedure was identical for both Experiment 1A and Experiment 1B with the only exception of the to-be-remember stimuli as mentioned above.

#### Data analysis

##### Availability

All data for the experiments are accessible on the Open Science Framework page associated with this project (**OSF**). Additionally, R markdown files for each experiment, including analysis and modelling codes, are also provided on the same page.

##### Scoring

In all experiments, a strict spelling criterion was applied. Recalled words were considered correct only if they were spelled accurately. For each experiment, we calculated the proportion of correct responses, omissions, intralist errors, false recalls of the critical lure, and extralist errors for each serial position. In addition, we present the item gradient for each position. The *proportion of correct responses* was determined using a strict serial recall criterion, where a word had to be recalled in its presented serial position to be deemed correct. An *omission* occurred when the participants either did not recall an item at a given serial position or typed a response indicating an omission (e.g., "skip", "unknown", etc.). The omissions were checked for each experiment by a research assistant who was blinded to the purpose of the experiment. An *intralist error* occurred when a presented word was recalled in a different position (e.g., a word presented in position 1 but recalled in position 2, 3, 4, 5, or 6) or was repeated (e.g., a word presented in position 1, but recalled twice in positions 2 and 3). The position uncertainty curves for each item were calculated by counting the proportion of time each word (e.g., word 1) was recalled in each serial position (1, 2, 3, 4, 5, 6) inspired by the seminal works of Estes (1991; Lee & Estes, 1977, 1981; Nairne 1991).

To assess false recall, we examined the specific critical lure and other extralist errors. A false recall was recorded as a critical lure when the recalled word corresponded to the lure associated with the immediately studied list. An extralist intrusion was defined as words recalled by participants that were not presented in the list, were not a critical lure of that list, and are included in the model's lexicon (41,006 words). For both types of false memory (critical lure and extralist error), only the first occurrence of a word was counted. More precisely, if a word was repeated (e.g., "bass, guitar, bass" in the same recall trial), only the first occurrence was considered.

##### Statistical analyses

All statistical analyses were conducted using the statistical software R (R Core Team, 2024), employing both frequentist and Bayes factor analyses. Our frequentist analyses were performed using the 'ez' package (version 4.4-0; Lawrence, 2016) for ANOVA. Our Bayes factor analyses were conducted with the 'BayesFactor' R package, utilizing the default priors (version 0.9.12-4.2; see Morey & Rouder, 2018; Rouder et al., 2009, 2012). These analyses involved 100,000 iterations, followed by an additional 10,000 iterations until the proportional error of the computation was reduced to less than 5%. Main effects and interactions in all Bayes factor ANOVAs were tested by omitting each effect from the full model, with participants included as a random factor (see Guitard et al., 2021, 2022; Guitard & Cowan, 2023 for similar procedures). For the Bayes factor analyses, we adopted the nomenclature where BF10 represents evidence for the alternative hypothesis and BF01 (BF01 = 1/BF10) indicates evidence for the null hypothesis. These procedures were conducted for each scoring procedure, considering serial position (1 to 6) as the only within-participants factor and experiment (related lists: Experiment 1A, unrelated list: Experiment 1B) as the only between-participants factor.

##### Simulation parameters

Two hundred participants were simulated, 100 simulations for Experiment 1A and 100 simulations for Experiment 1B. Due to the unavailability of the word “misfiling” in the model’s lexicon, “misfiling” was replaced by the word “filer” in our simulations. The same parameters were used for the simulation in Experiment 1A and Experiment 1B in which semantic representations were embedded in the eCFM: *L =* 0.265*, g =* 0.03*, d =* 0.3*, T =* 0.30*, s =* 0. For all the simulations, the simulations parameters are also presented in Table 1.

### Results

**Fig. 3:**
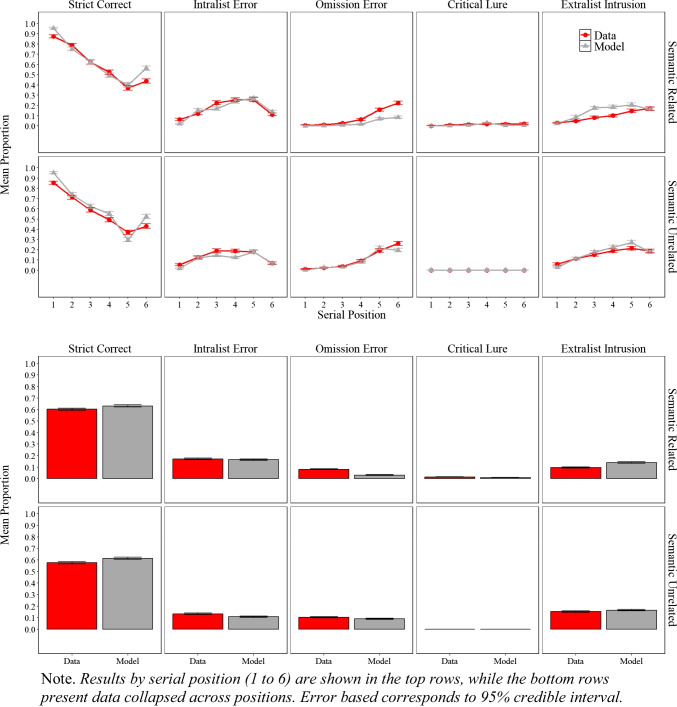
Model simulation results and experimental data for the mean proportion of correct recalls, intralist errors, omission errors, critical lure, and extralist error as a function of serial position in Experiment 1A (semantically related lists) and in Experiment 1B (semantically unrelated lists)

The experimental and simulation results of Experiment 1A and Experiment 1B are presented in Fig. 3, illustrating the proportion of correct responses, the proportion of intralist errors, omissions error and false memory as defined by critical lure and extralist intrusion as mentioned in the scoring section.

#### Experimental Results

In this section, we briefly present the main experimental results by comparing across experiments for each scoring procedure before presenting the model.

##### Proportion Correct

For the proportion of correct responses, participants’ performance was comparable between the related lists (*M* = .603, *SD* = .186) and the unrelated lists (*M* = .575, *SD* = .167), *F*(1,198) = 1.217*,* ${\eta }_{p}^{2}$ = .006, BF01 = 8.461. Although not the main theoretical focus, it is worth noting that the absence of a semantic similarity effect across our experiments appears inconsistent with the previously well-established semantic similarity advantage typically observed in within-participants manipulations (see Guitard et al., 2025; Neath et al., 2022, for a review). However, despite this general trend, there are some notable exceptions in which semantic similarity did not influence the overall recall performance. Unfortunately, these discrepant findings are often ignored to the benefit of the overall pattern. For instance, Baddeley (1966) reported a small but significant detrimental effect of semantic similarity on the proportion correct. Later, in their second experiment, using an immediate serial recall task, Saint-Aubin and Poirier (1999a) did not observe any benefit of semantic similarity on the proportion of correct recall. In addition, Poirier et al. (2015), reported that when all words in a list were related to a specific word, the semantic similarity advantage diminished, accompanied by an increase in intralist errors in the related conditions. This reduction in the advantage is likely due to the combined effect of increased intralist errors and a higher recall of critical lures in the semantically related condition, which constrained the distribution of errors and correct responses. As we previously argued, although necessary for comparisons across studies, the proportion of correct recall is not as informative as the distinct analysis of item and order recall reported in the section below (Saint-Aubin & Poirier, 1999b). Returning to the main analysis, as expected, we observed a main effect of serial position, reflecting the standard pattern: a primacy effect, with better recall of early-presented items, and a recency effect, with better recall of the last-presented items. *F*(5,990) = 430.068*,* ${\eta }_{p}^{2}$ = .685, BF10 > 10,000. There was no interaction between serial position and experiment (i.e., recall of semantically related versus semantically unrelated lists), *F*(5,990) = 2.026*,* ${\eta }_{p}^{2}$ = .010, BF01 = 90.328.

##### Intralist error

Participants in the related lists experiment made more intralist errors (M = .172, SD = .093) than participants in the unrelated lists experiment (M = .134, SD = .079), *F*(1,198) = 9.884*,* ${\eta }_{p}^{2}$ = .048, BF10 = 3.433. The analyses also revealed a main effect of serial position, *F*(5,990) = 115.454*,* ${\eta }_{p}^{2}$ = .368, BF10 > 10,000, and an interaction between serial position and experiment, *F*(5,990) = 5.400*,* ${\eta }_{p}^{2}$ = .027, BF10 = 589.272. The interaction is not of theoretical interest to the present investigation and reflects some minor differences in the number of intralist errors in later serial position across experiments.

##### Omission error

The number of omissions was comparable between the experiment with related lists (*M* = .082, *SD* = .110) and the experiment with unrelated lists (*M* = .103, *SD* = .120), *F*(1,198) = 1.640*,* ${\eta }_{p}^{2}$ = .008, BF01 = 6.258. The analysis also revealed the presence of a main effect of serial position, *F*(5,990) = 104.692*,* ${\eta }_{p}^{2}$ = .346, BF10 > 10,000, but no two-way interaction, *F*(5,990) = 0.521*,* ${\eta }_{p}^{2}$ = .003, BF01 = 770.881, between serial position and experiment.

##### Critical lure

As expected, participants were more likely to recall the specific critical lure with related lists (*M* = .015, *SD* = .016) relative to unrelated lists (*M* = .000, *SD* = .001), *F*(1,198) = 90.673*,* ${\eta }_{p}^{2}$ = .314, BF10 > 10,000. In addition, there was a main effect, of serial position, *F*(5,990) = 6.314*,* ${\eta }_{p}^{2}$ = .031, BF10 = 20.092, and a two-way interaction between serial position and experiment, *F*(5,990) = 6.638*,* ${\eta }_{p}^{2}$ = .032, BF10 = 256.182, reflecting the smaller increase of false memories in mid serial position for related lists.

##### Extralist error

Participants made more extralist errors with unrelated lists (*M* = .153, *SD* = .103) relative to related lists (*M* = .097, *SD* = .103), *F*(1,198) = 14.881*,* ${\eta }_{p}^{2}$ = .070, BF10 = 49.880. The analysis also revealed a main effect of serial position, *F*(5,990) = 59.243*,* ${\eta }_{p}^{2}$ = .230, BF10 > 10,000, and a two-way interaction between serial position and related/unrelated lists, *F*(5,990) = 6.638*,* ${\eta }_{p}^{2}$ = .017, BF10 = 65.238.

##### Summary experimental results

Overall, participants' performance was comparable in terms of the overall proportion of correct responses and the proportion of omissions. However, it differed with regard to intralist errors, with more errors associated with related lists than unrelated lists. More importantly, there was a higher proportion of false memories of the critical lures for related lists compared to unrelated lists. However, there were more extralist intrusions for unrelated lists compared to related lists. This reflects how the structure of the lists influences patterns of false memories. These results nicely confirm and extend the finding of Tehan (2010) in both immediate and delay serial recall. We now turn to an assessment of the model’s ability to track those outcomes.

#### Simulation results

##### Performance across experiments and serial positions

As shown in Fig. 3, the simulation results captured most of the key details, both at the overall level (lower panels) and as a function of serial position (upper panels). More exactly, for the proportion of correct responses, serial position functions show standard primacy and recency effects. For errors, simulations track the key details for intralist errors, such as fewer intralist errors at early serial positions with a minor discrepancy of predicting fewer intralist errors in the last serial position. The model tracks the main features of omission errors, with fewer omissions for early relative to late serial position, but produce fewer omissions in Experiment 1A relative to the data. Most importantly, the model produced false memories as defined by the critical lure and extralist intrusions at a similar rate to participants with the match between false recalls in the experiment and in the model simulations measured by precise word match (i.e., the model and people falsely recalled the same specific word rather than recalling a critical lure in principle) with only minor discrepancies, such as the model producing slightly more intrusions than we observed in the experimental data. Overall, across Experiment 1A, Experiment 1B, and the eCFM equipped with semantic word representations from LSA demonstrate an excellent fit to the empirical data, achieving an ${R}^{2}$= 0.97.

##### Positional uncertainty

**Fig. 4:**
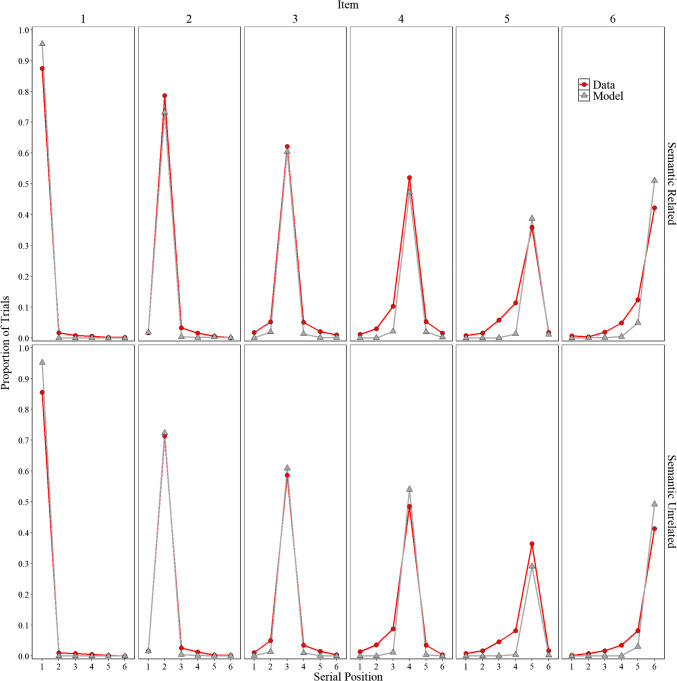
Model simulation results and experimental data for the proportion trials for each word (items 1–6) was recalled in each serial position (1–6) in Experiment 1A (semantically related lists) and in Experiment 1B (semantically unrelated lists)

Overall, the model represents an important initial step that allows for the direct comparison of participant behavior with that of a computational model. Before delving into specifics, we explore whether the implementation of order representation in Fig. 4, a novel feature in a model based on the MINERVA 2 (Hintzman, 1986) framework, can capture some aspects of the classic positional uncertainty curves. In line with Estes (1991, Lee & Estes, 1977, 1981) and Nairne (1991), the distributions are locality-constrained around the presented position, with more errors occurring at immediately adjacent serial positions than at serial positions farther away. We observe that the model mimics some of these features with errors more tightly clustered around adjacent positions compared to the data. There is still room for improvement, but the overall fit was good, achieving an ${R}^{2}$= 0.98. Importantly, these observations were not fitted; they are presented to illustrate some limitations while also highlighting potential directions for future research. We now return to our main focus, false memories.

##### Critical lure and extralist errors

**Fig. 5:**
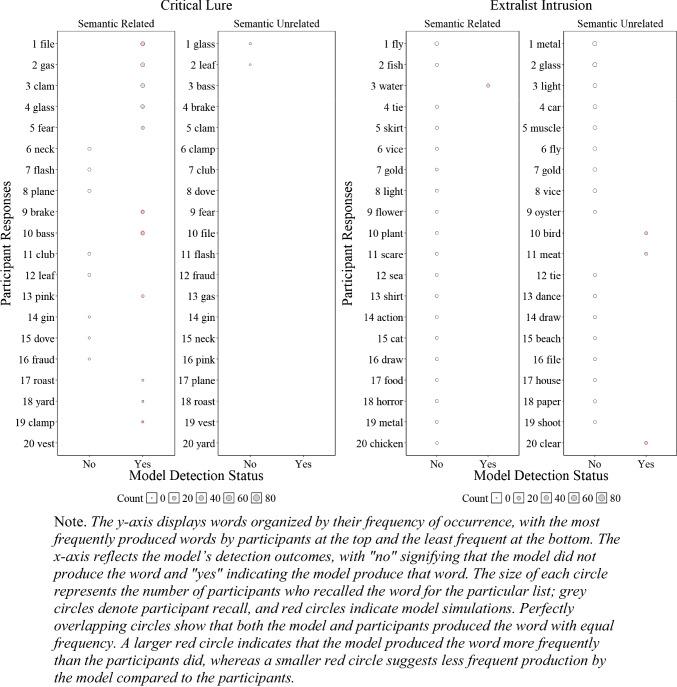
Illustration of the number of participant*s’* responses and number of model responses detections for the critical lure (**left panels**) and the 20 most common extralist intrusions collapsed across all lists (**right panels**) for Experiment 1A (semantically related words) and Experiment 1B (semantically unrelated words)

**Fig. 6:**
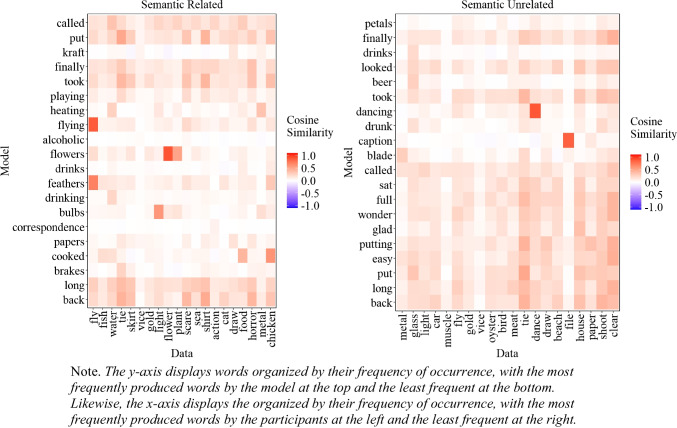
Cosine similarity matrix between the 20 most common extralist intrusions collapsed across all lists produced by the participants (x-axis) and the model (y-axis) for Experiment 1A (semantically related lists) and Experiment 1B (semantically unrelated lists)

Although the results presented in Fig. 6 and Fig. 4 are promising and represent a successful first step, having a lexicon allows us to further investigate whether the model accurately tracks specific false recalls reported by participants. To gain a deeper understanding of our ability to detect human memory errors, we examined at the item level, as shown in Fig. 5, whether the model tracked the specific critical lure and the 20 most common extralist intrusions that were available in the model’s lexicon. Readers who would like more information about the specific number of occurrences for each word are invited to consult the **OSF** page associated with this paper for each experiment.

Figure 5 illustrates whether the model was able to detect specific false memories and whether the frequency of these errors, represented by the size of the circles, was consistent with those of the participants. Overall, in Experiment 1A with semantically related lists, the model tracked 11 out of 19 produced critical lures with only minor divergences between the errors produced by the participants and the model, and 1 out of the 20 most common extralist errors, suggesting some deviation between the specificity of what the model and participants recalled at the item level—an insight not available without a structured lexicon. For semantically unrelated lists in Experiment 1B, the participants produced 2 out of 20 critical lures, and the model did not produce any, but the model tracked 3 out of the 20 most common extralist intrusions. Although these results might seem underwhelming, this is an important initial step as the overall predictions appear relatively accurate, but closer inspection has revealed important insights that additional work is needed to capture the precise specificity of human memory errors with semantically related materials.

##### Similarity between experimental data and model’s most common extralist errors

A natural question that arises is whether the most common extralist intrusions produced by the model are similar to those produced by participants, even though the model does not replicate the exact most common errors. To address this issue, we examined the cosine similarity matrix between the 20 most common extralist errors made by the participants and those generated by the model, aggregated across all lists, which is presented in Fig. 6. As Fig. 6 illustrates, in some instances, the most common responses of the participants matched those of the model, albeit with some words that are less related to the participants' most common responses (e.g., 'alcoholic'). Importantly, if the model behaved randomly, Fig. 6 would appear mostly white, corresponding to cosine values close to 0. The fact that we can capture some level of similarity between the most common errors of the participants and the model is an important initial step and provides some insight into the influence of semantic representation on memory performance.

##### Exploratory: Similarity between types of extralist errors in the data and the model

**Fig. 7:**
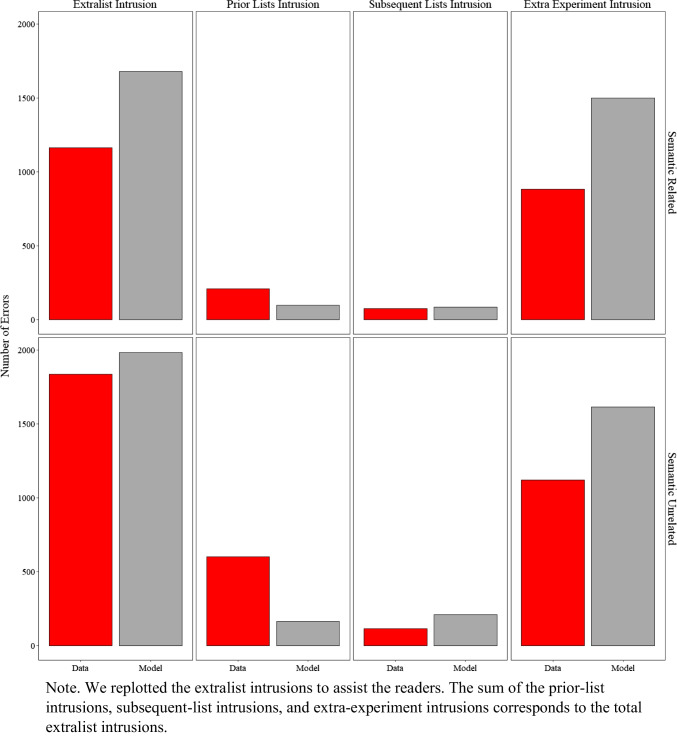
Model simulation results and experimental data for the number of extralist intrusions, prior list intrusions, subsequent list intrusions, and extra experiment intrusions in Experiment 1A (semantically related lists) and Experiment 1B (semantically unrelated lists)

In previous sections, we demonstrated the overall similarity between the specific and commonly occurring extralist errors produced by the model and those produced by participants. Based on a reviewer's recommendation, we now investigate the distribution of these extralist errors to more transparently highlight the strengths and limitations of the current approach, guiding areas for improvement in future work. Figure 7 presents the number of errors produced by participants and the model, categorized as prior-list intrusions, subsequent-list intrusions, and extra-experiment intrusions.

A prior-list intrusion involves a word presented in any lists before the current one but not in the current list (e.g., the word *cat* recalled on list *n* but presented on list *n–1*). A subsequent-list intrusion involves a word presented later in the experiment but not before the current list (e.g., the word *cat* recalled on list *n* but presented on list *n+1*). An extra-experiment intrusion involves a word neither presented in the experiment, nor a critical lure.

Overall, the model fit the data well (R^2^ = 0.85), with minor and expected discrepancies. As shown in Fig. 7, the total number of extralist intrusions was relatively comparable between participants and the model, though the model slightly overproduced these errors. Examining the distribution of errors, extra-experiment intrusions were the most frequent type, which the model also slightly overproduced. Prior-list intrusions occurred more often in the data than subsequent-list intrusions, a pattern more pronounced in the unrelated lists from Experiment 1B. This difference is likely because participants tested with related lists in Experiment 1A produced more critical lures, which were more strongly activated in memory than prior-list items.

Due to the trial-unit nature of the current model—where memory is reset after each trial—it was unable to capture this subtle difference. A discussion of potential model extensions to address these limitations is deferred to the general discussion.

### Discussion

In Experiments 1A and 1B, we aimed to examine whether the eCFM, by embedding a lexicon that contains semantic relationships among words, could capture false memories with both semantically related and unrelated materials while tracking memory performance across key metrics. Overall, the model does a good job of capturing memory performance across various measures, such as proportion correct, intralist errors, omissions, and false memories of the critical lure and non-critical extralist intrusions. Although the model produces a relatively good fit to the data at the global level, we were able to further investigate at the item level due to the embedded lexicon. Overall, at the item level, there was some initial success in detecting word specific false recalls.

## Phonological: Experiment 2A & Experiment 2B

Experiments 1A and 1B provided clear evidence of the benefits of embedding a lexicon to capture semantic relationships in accounting for false memories involving semantically related and unrelated materials. In Experiments 2A and 2B, our goal was to investigate the flexibility of the model in capturing phonologically related false recalls. This was achieved by altering the study lists and substituting the model's lexicon of semantic word representations (Landauer & Dumais, 1997) with a lexicon of phonological word representations (Parrish, 2017). Like Experiment 1A and Experiment 1B, we present Experiment 2A that tested people’s serial recall for related lists and Experiment 2B that tested people’s serial recall for unrelated lists together, to facilitate understanding of the key empirical and simulation findings.

### Method

#### Participants

The sample size justification and inclusion criteria were identical to Experiment 1, with the additional condition that participants from Experiment 1A and Experiment 1B were excluded from participating in these experiments. Therefore, another 200 participants were recruited via Prolific. In Experiment 2A, the participants had a mean age of 22.58 years (*SD* = 2.04). Of these, 52 self-identified as female, 45 as male, and 3 chose not to specify their gender. In Experiment 2B, the participants had a mean age of 22.39 years (*SD* = 1.99). Among them, 58 self-identified as female and 42 self-identified as male.

#### Materials

In Experiments 2A, like in Experiments 1A, a total of 20 lists were employed, each comprising six words phonologically related to an unpresented critical lure. For this experiment, the phonological study lists were curated using the English Lexicon Project (Balota et al., 2007). Specifically, we selected 20 one-syllable target words, varying from 3 to 6 letters in length, along with their corresponding phonological neighbors. In Experiment 2B, like Experiment 1B, the words were arranged to minimize the similarity among the words. The specific lists used in both experiments are presented in Appendix B, along with the mean cosine similarity among the words and the critical lure for each list. The average cosine similarity across all lists in Experiment 2A was higher (*M* = .376, *SD* = .059) than that in Experiment 2B (*M* = .162, *SD* = .020), as revealed by a Bayesian t-test, with a Bayes factor (BF10) greater than 10,000.

#### Procedure and data analysis

The experimental procedure and data analysis methods in Experiments 2A and 2B matched those of Experiments 1A and 1B, except for the stimuli. In this experiment, words in the same list as well as their critical lure were phonologically rather than semantically related in Experiment 2A and phonologically rather than semantically unrelated in Experiment 2B.

#### Simulation parameters

Like Experiments 1A and 1B, 200 simulations, 100 simulations for Experiment 2A and 100 simulations for Experiment 2B, were conducted with the eCFM. All parameters were identical to Experiment 1A and 1B except for the embedded representations (see Table 1), which were changed to the phonological representations to reflect a phonological encoding strategy and the learning rate which was set slightly lower: *L =* 0.215*, g =* 0.03*, d =* 0.3*, T =* 0.30*, s =* 0.

### Results

**Fig. 8:**
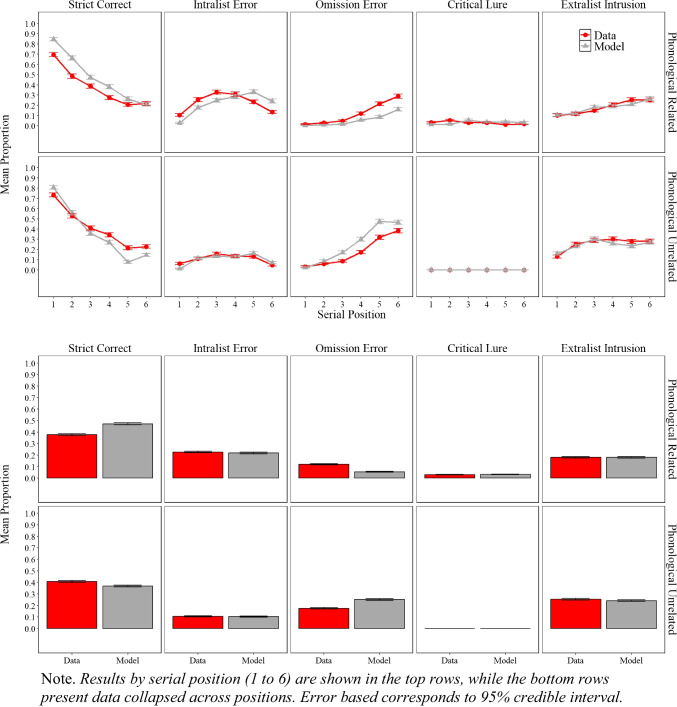
Model simulation results and experimental data for the mean proportion of correct recalls, intralist errors, omission errors, critical lure, and extralist error as a function of serial position in Experiment 2A (phonologically related lists) and in Experiment 2B (phonologically unrelated lists)

Figure 8 shows the experimental and simulation results of Experiment 2A with related lists and Experiment 2B with unrelated lists, illustrating the proportion of correct responses, the proportion of intralist errors, omission errors, and false recalls for the critical lures and noncritical extralist intrusions.

#### Experimental results

Like Experiments 1A and 1B, we first summarize the main findings from each scoring method across Experiments 2A and 2B before presenting the model.

##### Proportion correct

Participants’ performance was comparable in the unrelated lists experiment (*M* = .409, *SD* = .191) compare to the related lists experiment (*M* = .378, *SD* = .173), *F*(1,198) = 1.476*,* ${\eta }_{p}^{2}$ = .005, BF01= 7.125. Although numerically consistent with the classic within-participants detrimental effect of phonological similarity, our experiments do not exhibit the large detrimental effect of similarity typically observed in other phonological similarity manipulation studies (see Roodenrys et al., 2022, for a review). Once more, this pattern has also been observed in the past (e.g., Fallon et al., 1999, 2005). Most importantly, this pattern is not theoretically consequential, as it likely reflects a redistribution of correct and error responses when words are related to a specific target. This phenomenon has also been observed by Saint-Aubin et al. (2023), who reported that when words were orthographically and phonologically related to a specific item, the expected detrimental effect was reduced. However, in line with the previous experiments there was a main effect of serial position, *F*(5,990) = 455.546*,* ${\eta }_{p}^{2}$ = .697, BF10 > 10,000, and no interaction between serial position and list type, *F*(5,990) = 1.590*,* ${\eta }_{p}^{2}$ = .008, BF01 = 662.525.

##### Intralist error

Like in the previous experiments, there were more intralist errors in the related lists experiment (M = .228, SD = .094) relative to the unrelated lists experiment (M = .107, SD = .070), *F*(1,198) = 106.235*,* ${\eta }_{p}^{2}$ = . 349, BF10 >10,000. There was also a main effect of serial position, *F*(5,990) = 85.079*,* ${\eta }_{p}^{2}$ = .301, BF10 > 10,000, and an interaction between serial position and experiment, *F*(5,990) = 13.030*,* ${\eta }_{p}^{2}$ = .062, BF10 > 10,000, with a more pronounced rate of intralist errors in the middle serial positions for related lists.

##### Omission error

There were more omissions in the unrelated lists experiment (*M* = .176, *SD* = .176) compare to the related lists experiment (*M* = .120, *SD* = .170), *F*(1,198) = 5.262*,* ${\eta }_{p}^{2}$ = .026, but the evidence was inconclusive, BF10 = 1.173. The results from the analysis show evidence in favor of a main effect of serial position, *F*(5,990) = 123.435*,* ${\eta }_{p}^{2}$ = .384, BF10 > 10,000, and a two-way interaction, *F*(5,990) = 2.473*,* ${\eta }_{p}^{2}$ = .012, BF10 > 10,000.

##### Critical lure

As expected and like in the previous experiments, there were more critical lure errors in the related lists experiment (*M* = .030, *SD* = .017) compared to unrelated lists experiment (*M* = .000, *SD* = .002), *F*(1,198) = 287.996*,* ${\eta }_{p}^{2}$ = .593, BF10 > 10,000. There was also a main effect of serial position, *F*(5,990) = 13.260*,* ${\eta }_{p}^{2}$ = . 063, BF10 > 10,000, and an interaction between serial position and experiment, *F*(5,990) = 13.260*,* ${\eta }_{p}^{2}$ = .063, BF10 > 10,000.

##### Extralist error

Like the previous experiments, extralist errors were more common in the unrelated lists experiment (*M* = .256, *SD* = .160) than the related lists experiment (*M* = .180, *SD* = .094), *F*(1,198) = 12.644*,* ${\eta }_{p}^{2}$ = .060, BF10 = 23.178. There was also a main effect of serial position *F*(5,990) = 44.252*,* ${\eta }_{p}^{2}$ = . 183, BF10 > 10,000, and an interaction between serial position and experiment, *F*(5,990) = 9.456*,* ${\eta }_{p}^{2}$ = .046, BF10 > 10,000.

##### Summary experimental results

Similar to Experiments 1A and 1B, the proportion of correct responses showed that participant performance was relatively similar across experiments. However, there were more intralist errors in the experiment with related lists compared to the experiment with unrelated lists; a result that is consistent with numerous findings on memory for phonologically related lists (e.g., Roodenrys et al., 2022, for a review and empirical results). The number of omissions was comparable between the related lists and the unrelated lists. Importantly, for the present study, there was a higher incidence of false memories related to the critical lures in the related lists compared to the unrelated lists, and fewer extralist intrusions in the related lists compared to the unrelated lists. Overall, our empirical results nicely extend the findings of Tehan (2010) with semantic material to phonological materials. In the next section, we explore the eCFM’s ability to track these outcomes.

#### Simulation results

##### Performance across experiments and serial positions

As shown in Fig. 8, the simulation results captured many key empirical findings at both the overall level (bottom panels) and as a function of serial position (upper panels). The eCFM accurately captured empirical results, including features such as correct responses, proportion correct for early versus later serial positions, intralist errors, along with critical lure and extralist intrusions. However, there were some challenges in accurately capturing certain features, such as slightly overpredicting performance for related lists and underpredicting performance for unrelated lists in terms of proportion correct and omissions, as well as producing more intralist errors in late serial positions (5 and 6) for related lists. Again, and more important for the present study that incorporates a lexicon of phonological rather than semantic word representations, the model produced false recalls at a rate comparable to that of the participants with only minor divergence. In summary, the eCFM, across Experiment 2A and Experiment 2B, equipped with phonological representations, provides a reasonably good fit to the empirical data, achieving an ${R}^{2}$= 0.89.

##### Positional uncertainty

**Fig. 9:**
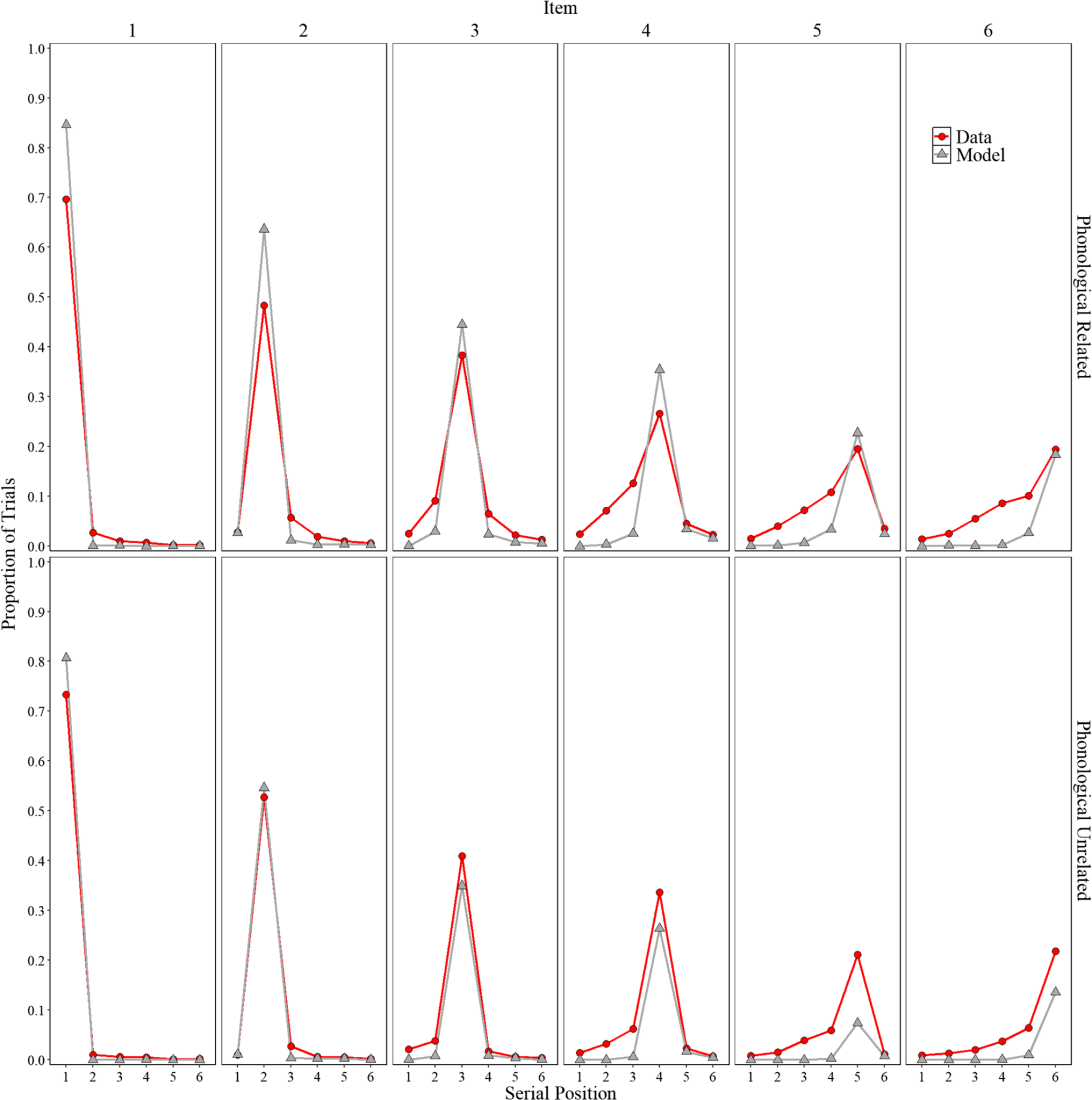
Model simulation results and experimental data for the proportion trials for each word (item 1–6) was recalled in each serial position (1–6) in Experiment 2A (phonologically related lists) and in Experiment 2B (phonologically unrelated lists)

We again briefly examined the positional uncertainty curves in Fig. 9, this time with phonological representations. Like Experiments 1A and 1B, the distributions are locality-constrained around the presented position, with more errors occurring at immediate and near adjacent positions than at positions farther away. Overall, the fit was good, achieving an ${R}^{2}$= 0.95. However, the model often shows more clustering around adjacent positions compared to the empirical data, which exhibits a gentler slope across adjacent positions.

##### Critical lure and extralist errors

**Fig. 10:**
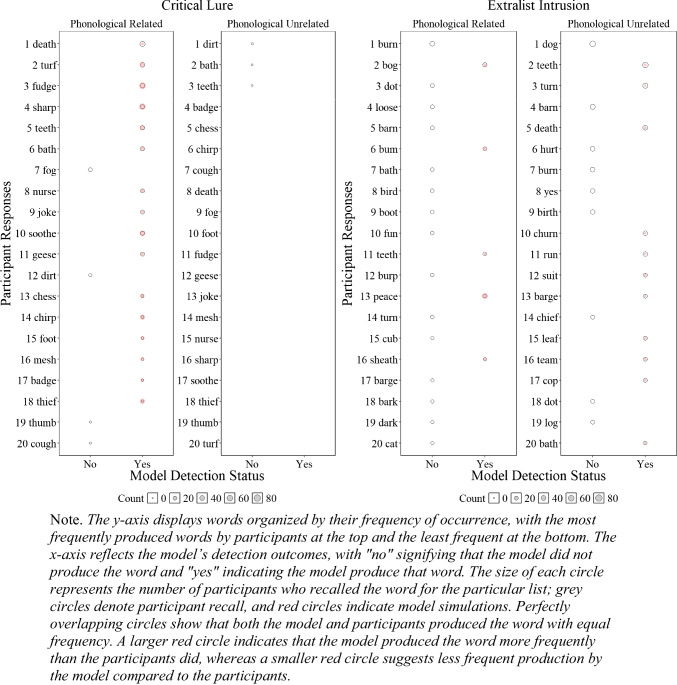
Illustration of the number of participants’ responses and number of model responses detections for the critical lure (**left panels**) and the 20 most common extralist intrusions collapsed across all lists (**right panels**) for Experiment 2A (phonologically related words)

Although the initial results presented in Fig. 8 and 9 are promising and represent an important first step at model evaluation, having a lexicon allows for an additional level of investigation, like in Experiments 1A and 1B. As shown in Fig. 10, we examined whether the model accurately tracked the specific critical lures people recalled and the 20 most common and noncritical extralist intrusions available in the model’s lexicon. Figure 9 shows whether the model was able to detect specific false memories and whether the frequency of these errors, represented by the size of the circles, was consistent with those of the participants. Overall, in Experiment 2A with phonologically related lists, the model tracked 16 out of 20 produced critical lures with only minor divergences in terms of frequency, with some instances where the model overproduced or underproduced false recall of a specific word. For unrelated lists, participants produced 3 of the 20 critical lures at a low frequency, and this idiosyncratic property of the experimental results was not captured by the model. For the extralist errors in the related lists experiment, the model captured 5 out of the 20 most common extralist errors, and 11 out of 20 for the unrelated lists. This level of precision, although not perfect, is an important demonstration of the value of having a lexicon capturing phonological representations.

##### Similarity between experimental data and the model’s most common extralist errors

**Fig. 11:**
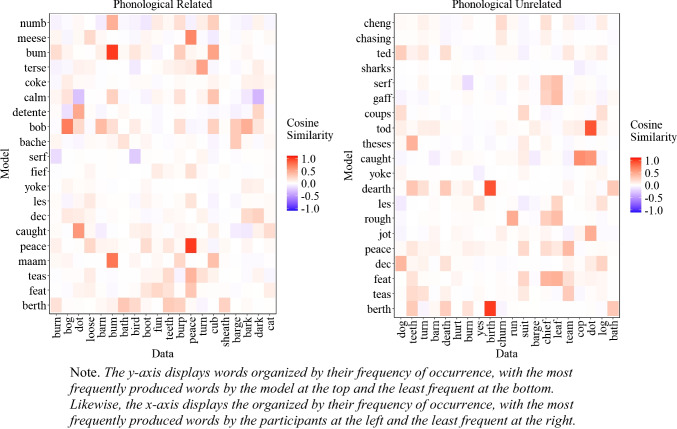
Cosine similarity matrix between the 20 most common extralist intrusions collapsed across all lists produced by the participants (x-axis) and the model (y-axis) for Experiment 2A (phonologically related lists) and Experiment 2B (phonologically unrelated lists)

Like in previous experiments, we examined the similarity between the most common extralist intrusions produced by the model and those produced by the participants. To visualize and explore that comparison, we present the cosine similarity matrix in Fig. 11 between the 20 most common extralist errors made by participants (x-axis) and those generated by the model (y-axis), aggregated across all lists. As shown, some of the participants’ most common responses matched those of the model identically, and some shared phonological features (e.g., ‘dog’ and ‘tod’), while others were more distinct (e.g., ‘yoke’). Importantly, the model produced many responses similar to those of the participants, providing additional evidence of the influence of embedding phonological representations on predicting memory performance.

##### Exploratory: Similarity between types of extralist errors in the data and the model

**Fig. 12:**
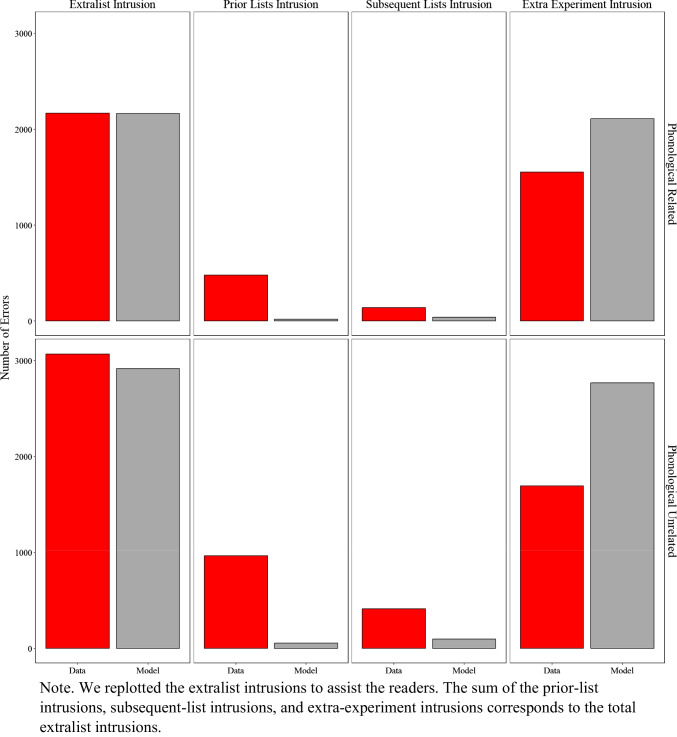
Model simulation results and experimental data for the number of extralist intrusions, prior list intrusions, subsequent list intrusions, and extra experiment intrusions in Experiment 2A (phonologically related lists) and in Experiment 2B (phonologically unrelated lists)

As in Experiment 1, we analyzed the distribution of extralist errors presented in Fig. 12, which categorizes the errors made by participants and the model into prior-list intrusions, subsequent-list intrusions, and extra-experiment intrusions.

Overall, the model fits the data reasonably well (R^2^ = 0.81), with only minor and expected discrepancies. For instance, prior-list intrusions were more pronounced in the data compared to the model. As shown in Fig. 12, the total number of extralist intrusions was comparable between participants and the model. Consistent with Experiments 1A and 1B, extra-experiment intrusions were the most frequent type of error, which the model once again slightly overproduced. Prior-list intrusions occurred more frequently in the data than subsequent-list intrusions, a pattern more evident in the unrelated list experiment. This discrepancy likely arises because participants in the related condition produced more critical lures, as these lures were more strongly activated than items from prior lists. As expected and consistent with earlier experiments, the model produced similar rates of prior and subsequent intrusions, highlighting a current limitation of this approach.

### Discussion

When studying phonologically related lists in Experiment 2A, participants recalled the critical unpresented words more than when participants in Experiment 2B studied unrelated lists. More critically, our simulations with the eCFM demonstrate that embedding a lexicon into a memory model enables the generation of phonologically-related false memories, while also accurately replicating many key features across various memory measures, such as the proportion of correct responses and better recall for words presented early in the list. More exactly, upon closer examination of specific items, the model was able to capture nearly all the critical lures for related lists and more than half of the extralist errors for the unrelated experiment. This detailed exploration is made possible by integrating a lexicon into an episodic memory account of serial recall to capture phonological relationship between the words in the lexicon.

## Orthographic: Experiment 3A & Experiment 3B

Our previous experiments provide evidence supporting the value of embedding a lexicon to capture both semantic and phonological false memories. In this experiment, our goal was to investigate the model's flexibility in capturing orthographically related non-word false memories with orthographically related lists in Experiment 3A and orthographically unrelated lists in Experiment 3B. This was achieved by altering the study lists and substituting the model's lexicon with non-word representations inspired by the open-bigram scheme from SERIOL and SERIOL2 (Whitney, 2001; Whitney & Marton, 2013). Like previous experiments, Experiment 3A with related lists and Experiment 3B with unrelated lists are presented together to facilitate understanding of the key empirical and simulation findings.

### Method

#### Participants

The sample size justification and inclusion criteria for Experiments 3A and 3B remained consistent with our previous experiments. However, participants who had taken part in the previous experiments were excluded from participating in Experiments 3A and 3B.

Thus, an additional 200 participants were recruited through Prolific. The 100 participants in Experiment 3A had an average age of 22.53 years (*SD* = 1.72). Of these participants, 67 self-identified as female, 30 as male, and 3 chose not to specify their gender. The 100 participants in Experiment 3B had an average age of 22.47 years (*SD* = 2.12). Of these participants, 59 self-identified as female, 37 as male, and 4 chose not to specify their gender.

#### Materials

In Experiment 3A, as in Experiments 1A and 2A, a total of 20 related lists were constructed. Each list consisted of six three-letter non-words that were orthographically related to one another and to an unpresented critical lure. Because all non-words were three-letter consonant strings (i.e., no vowels including “sometimes y”), we were confident that none of the items was a word. However, to ensure the exclusion of real words, these non-words were carefully examined by the experimenter who also confirmed their absence in the English Lexicon Project (Balota et al., 2007). In Experiment 3B, the non-words were arranged to minimize the similarity among the non-words. The specific non-words used for each experiment are presented in Appendix C, along with the mean cosine similarity among the non-words and the critical lure for each list. The average cosine similarity across all lists in Experiment 3A was superior (*M* = .501, *SD* = .011) compared to that in Experiment 3B (*M* = .217, *SD* = .020), as revealed by a Bayesian *t*-test, with a Bayes factor (BF10) greater than 10,000. Like the preceding experiments, the item sequence within each list remained constant, but the order of presentation for the 20 lists was randomized for each participant.

#### Procedure and data analysis

The experimental procedure and data analysis methods in Experiments 3A and 3B were identical to those of previous experiments, with the exception of the memoranda. In these experiments, the stimuli consisted of non-words that were orthographically related in Experiment 3A and orthographically unrelated Experiment 3B.

#### Simulation parameters

Like previous experiments, 200 simulations, 100 simulations for Experiment 3A and 100 simulations for Experiment 3B, were conducted with the eCFM using the same word lists from the experiment. All parameters were identical to Experiment 2A and 2B except for the embedded representations (see Table 1), which were changed to the orthographic representations to reflect the features of the task and the learning rate which was set slightly lower: *L =* 0.19*, g =* 0.03*, d =* 0.3*, T =* 0.30*, s =* 0.

### Results

**Fig. 13:**
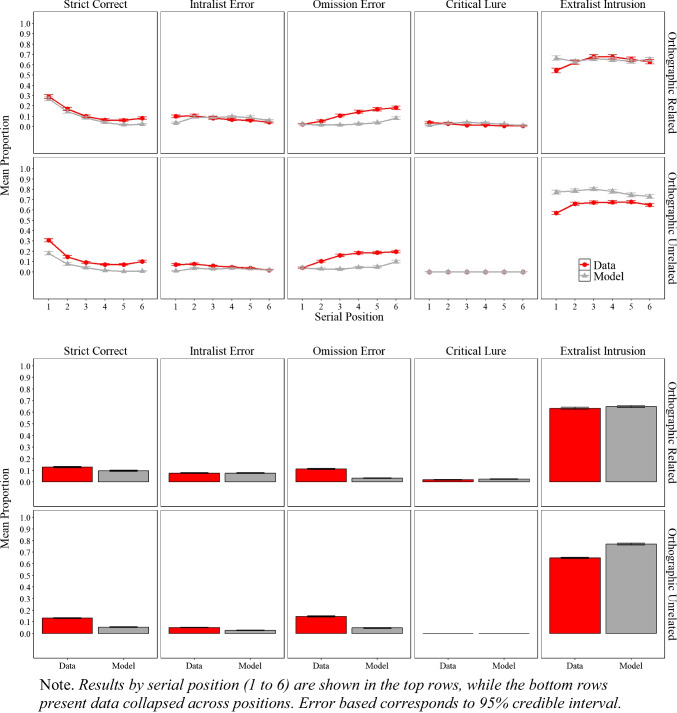
Model simulation results and experimental data for the mean proportion of correct recalls, intralist errors, omission errors, critical lure, and extralist error as a function of serial position in Experiment 3A (orthographically related lists) and in Experiment 3B (orthographically unrelated lists)

Figure 13 presents the experimental and simulation results from Experiment 3A, which used related lists, and Experiment 3B, which used unrelated lists. It illustrates the proportion of correct responses, the proportion of intralist errors, omission errors, and false memories for both the critical items and the extralist intrusions.

#### Experimental results

Like previous experiments, we first summarize the main findings from each scoring method across Experiments 3A and 3B before presenting results from the model.

##### Proportion correct

The performance of participants in the unrelated lists experiment (*M* = .131, *SD* = .159) was similar to the performance of participants in the experiment with the related lists (*M* = .128, *SD* = .146), *F*(1,198) = 0.015*,* ${\eta }_{p}^{2}$ = .000, BF01 = 11.914. These results are consistent with our previous findings, which showed no credible difference between related and unrelated experiments, and align with prior studies in which words were organized to be related to a key item (e.g., Saint-Aubin et al., 2023). Again there was a main effect of serial position, *F*(5,990) = 163.539*,* ${\eta }_{p}^{2}$ = . 452, BF10 > 10,000, but no interaction, *F*(5,990) = 1.455*,* ${\eta }_{p}^{2}$ = .007, BF01 = 33.646.

##### Intralist error

Once more, the proportion of intralist errors in the related lists experiment (*M* = .076, *SD* = .076) was to superior to that in the unrelated lists experiment (*M* = .052, *SD* = .050), but the difference was not statistically reliable, *F*(1,198) = 7.082*,* ${\eta }_{p}^{2}$ = . 035, BF10 = 1.259. The results from the analyses also confirmed a main effect of serial position, *F*(5,990) = 22.362*,* ${\eta }_{p}^{2}$ = .101, BF10 > 10,000, and the absence of interaction between serial position and experiment, *F*(5,990) = 0.072*,* ${\eta }_{p}^{2}$ = .000, BF01 > 10,000.

##### Omission error

The proportion of omissions was comparable between the unrelated lists experiment (*M* = .145, *SD* = .253) and the related lists experiment (*M* = .112, *SD* = .217), *F*(1,198) = 0.977*,* ${\eta }_{p}^{2}$ = .005, BF01 = 4.105. Again there was a main effect of serial position, *F*(5,990) = 40.925*,* ${\eta }_{p}^{2}$ = .171, BF10 > 10,000, and some evidence in favor of a two-way interaction, *F*(5,990) = 0.651*,* ${\eta }_{p}^{2}$ = .003, BF10 = 20.803.

##### Critical lure

In line with the previous experiments, participants recalled the critical lure in the related lists experiment (*M* = .019, *SD* = .016) more often than participants in the unrelated lists experiment (*M* = .000, *SD* = .000), *F*(1,198) = 127.339*,* ${\eta }_{p}^{2}$ = .391, BF10 > 10,000. The analyses also revealed the main effect of serial position, *F*(5,990) = 14.010*,* ${\eta }_{p}^{2}$ = . 066, BF10 > 10,000, and the interaction between serial position and experiment*, F*(5,990) = 14.010*,* ${\eta }_{p}^{2}$ = . 066, BF10 > 10,000.

##### Extralist error

In this experiment, the proportions of extralist errors were comparable between the unrelated lists experiment (*M* = .652, *SD* = .265) and the related lists experiment (*M* = .634, *SD* = .265), *F*(1,198) = 0.215*,* ${\eta }_{p}^{2}$ = .001, BF01 = 8.058. A main effect of serial position was observed, *F*(5,990) = 19.599*,* ${\eta }_{p}^{2}$ = .090, BF10 > 10,000, with no evidence of a statistically significant interaction between serial position and experiment, *F*(5,990) = 0.630*,* ${\eta }_{p}^{2}$ = .003, BF01 > 10,000.

##### Summary experimental results

As in previous experiments, the proportion of correct responses was comparable across experiments with both related and unrelated lists. The proportion of intralist errors, omissions, and extralist errors was similar between the experiments involving related and unrelated lists defined by semantic, phonological, and orthographic similarity. However, in line with our previous findings and as expected, there were more false memories related to the critical lures in the related lists compared to the unrelated lists. These results nicely extend the findings of Tehan (2010), who studied semantic material, to orthographic non-words materials. We now investigate the model's ability to track these findings.

#### Simulation eesults

##### Performance across experiments and serial positions

As illustrated in Fig. 15, the eCFM effectively captured many key features of the findings both overall and across serial positions. The eCFM accurately accounts for many empirical results, capturing aspects such as correct responses, a slight advantage for early versus later serial positions, intralist errors, as well as critical lure and extralist intrusions. However, there were some challenges in accurately predicting the pattern of omissions in both related and unrelated lists, with the model underestimating the number of omission errors and slightly overpredicting the rate of extralist intrusions—such as at serial position 1 for the related lists experiment and across most serial positions for the unrelated experiment. Importantly, for the current study that incorporates a lexicon of orthographic representations, the model produced false recalls at a rate comparable or slightly greater than participants. In summary, the eCFM, equipped with orthographic representations, provides a reasonably good fit to the empirical data on both accurate and false recall of orthographically related lists, achieving an ${R}^{2}$= 0.95.

##### Positional uncertainty

Consistent with prior findings, the distributions are clustered around the studied serial position, though with some minor discrepancies. For example, the model underestimated the proportion of trials in which items 5 and 6 were recalled in their presented positions. However, the model once again captured many important features, such as the expected decrease across adjacent positions for items at serial positions 2 and 3. The fit was good, but poorer compared to the previous experiment, ${R}^{2}$= 0.86.

##### Critical lure and extralist errors

**Fig. 14:**
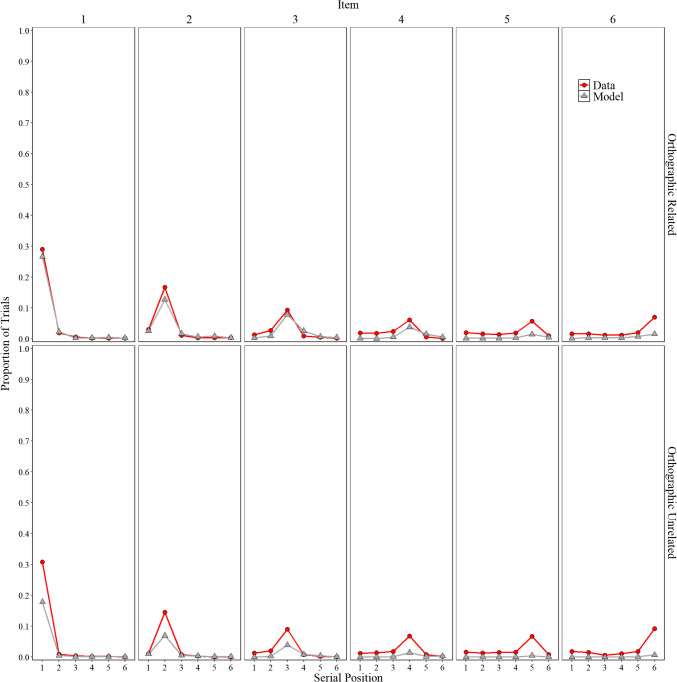
Model simulation results and experimental data for the proportion trials for each word (item 1–6) was recalled in each serial position (1–6) in Experiment 3A (orthographically related lists) and in Experiment 3B (orthographically unrelated lists)

**Fig. 15:**
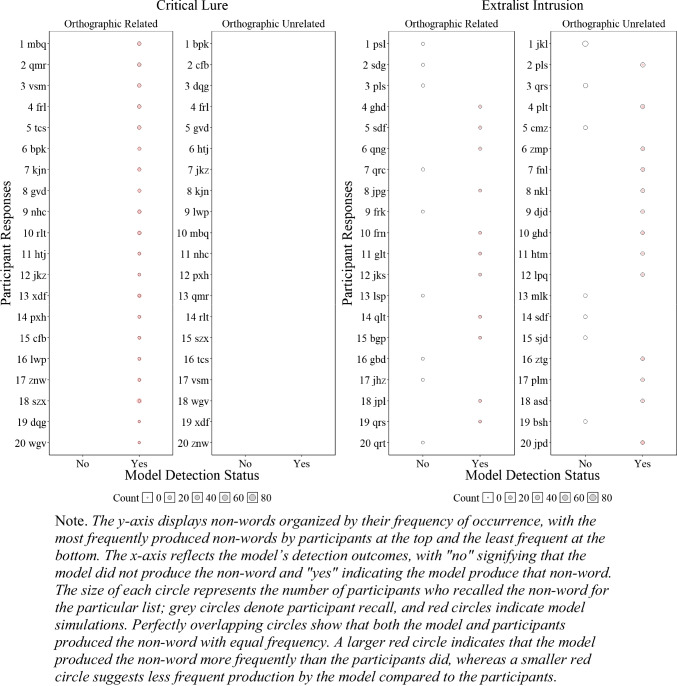
Illustration of the number of participants’ responses and number of model responses detections for the critical lure (**left panels**) and the 20 most common extralist intrusions collapsed across all lists (**right panels**) for Experiment 3A (orthographically related non-words) and Experiment 3B (orthographically unrelated non-words)

The results presented in Fig. 13 and 14 are promising and demonstrate the model's ability to track the overall pattern of memory performance. However, as in previous experiments, the integration of a lexicon into our model enables us to explore word-level predictions, which are presented in Fig. 15.

Figure 15 shows the extent to which the model accurately tracked the specific critical lures and the 20 most common extralist intrusions contained within the model’s lexicon. As shown, the model successfully detected all specific critical lures at a frequency comparable to that observed in the related lists experiment, and neither the model nor the participants produced any of these critical lures in the unrelated experiment. For the extralist errors in the related lists experiment, the model captured 11 out of the 20 most common extralist errors, and 13 out of 20 for the unrelated lists. This level of precision provides further evidence of the value of including a lexicon for precise prediction; even for the very non-wordy 3-letter consonant strings that we used in our experiment.

##### Similarity between experimental data and model’s most common extralist errors

**Fig. 16:**
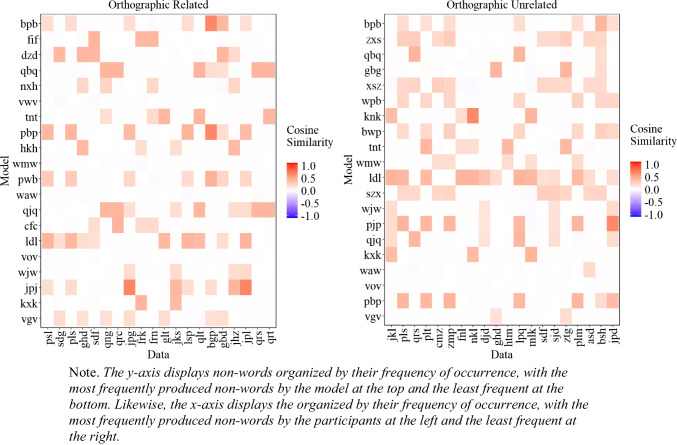
Cosine similarity matrix between the 20 most common extralist intrusions collapsed across all lists produced by the participants (x-axis) and the model (y-axis) for Experiment 3A (orthographically related lists) and Experiment 3B (orthographically unrelated lists)

In this section, as in the previous experiments, we examined the similarity between the most common extralist intrusions produced by the model and those produced by participants. The results are presented in the cosine similarity matrix between the 20 most common extralist errors made by the participants and those generated by the model, aggregated across all lists in Fig. 16. If there were no overlap between the participants and the model, the cosine similarity matrix would be completely white. However, we can clearly see that almost all the most common responses of the participants shared many orthographic features with at least one response of the model (e.g., 'pbp' and 'jpj'), while a few responses of the model were more distinct (e.g., 'vow').

##### Exploratory: Similarity between types of extralist errors in the data and the model

**Fig. 17:**
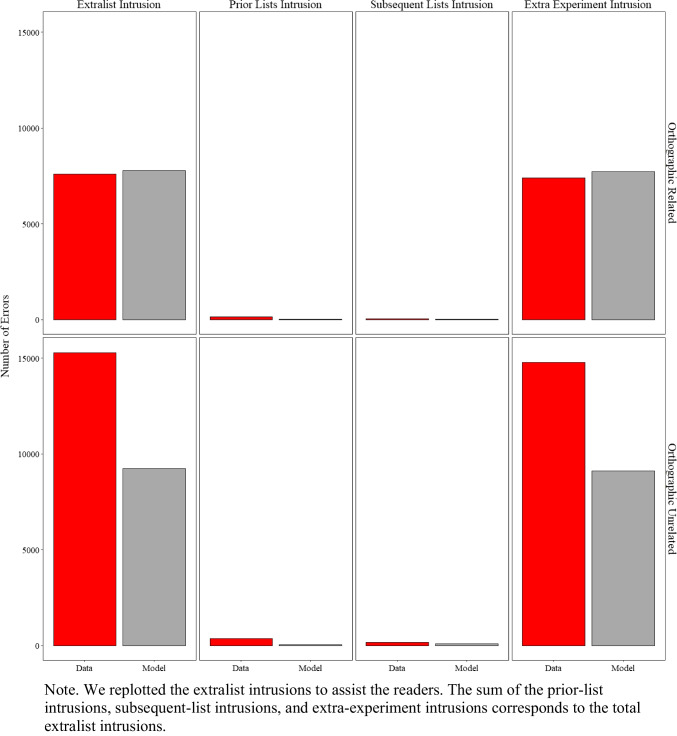
Model simulation results and experimental data for the number of extralist intrusions, prior list intrusions, subsequent list intrusions, and extra experiment intrusions in Experiment 3A (orthographically related lists) and Experiment 3B (orthographically unrelated lists)

We further examined the distribution of extralist errors, as presented in Fig. 17. This figure illustrates the number of errors made by participants and the model, categorized into prior-list intrusions, subsequent-list intrusions, and extra-experiment intrusions.

Overall, the model performed reasonably well (R^2^ = 0.90). As shown in Fig. 17, the total number of extralist intrusions and extra-experiment intrusions was comparable between participants and the model in the related experiment but was underproduced by the model in the unrelated experiment. Consistent with previous experiments, prior-list intrusions were more frequent in the data than subsequent-list intrusions, with this pattern being more pronounced in the unrelated experiment than related experiment. However, these errors were relatively rare compared to extra-experiment intrusions. As expected, and consistent with earlier findings, the model produced similar rates of prior and subsequent intrusions but failed to capture this particular feature of the data.

### Discussion

With orthographically related lists in Experiment 3A, participants were more likely to recall critical unpresented words compared to participants in Experiment 3B who studied unrelated lists. However, in both experiments, participants produced a larger number of extralist intrusions. More importantly, our simulations with the eCFM demonstrate that embedding a lexicon that integrates orthographic representations in a model of episodic memory enables the generation of false memories for both orthographically related and unrelated lists. Additionally, it captures key features across various memory measures, such as superior memory performance for early non-words in the list. Pertinent to this study, when examining specific items, the model successfully captured all the critical lures for related lists and more than half of the extralist errors for both related and unrelated experiments. Overall, this provides clear evidence that embedding a lexicon into a memory model enhances the operative depth of predictive precision.

## Semantic: Simulation full model

The results of our previous six experiments are clear and provide evidence of the benefits of embedding a lexicon into a memory model to capture memory performance for semantically (Experiments 1A, 1B), phonologically (Experiments 2A, 2B), and orthographically (Experiments 3A, 3B) related versus unrelated materials. One potential caveat is that our earlier simulations employed a lexicon tailored to the experiment materials—for instance, only semantic representations were used for simulating semantically related versus semantically unrelated lists in Experiments 1A and 1B. Although there is growing evidence that participants can attend to specific features of memoranda based on task demands and materials in both serial recall (e.g., Guitard et al., 2021, 2022, 2023) and recognition (e.g., Caplan, 2023; Caplan & Guitard, 2024a, b), it is indisputable that memory performance is influenced by semantic, phonological, and orthographic characteristics in tandem (e.g., Guitard & Cowan, 2020; Neath et al., 2022; Roodenrys et al., 2022). For example, people might falsely recall *puff* after studying *tough* (phonological) and falsely recall *car* after studying *truck* (semantic) within the same recall trial. To address this fact, we implemented a comprehensive model that integrates orthographic, phonological, and semantic representations. We examined whether this enhanced lexicon could still track the performance of semantically related and unrelated materials.

### Method

#### Simulation parameters

All simulation details were identical to Experiments 1A and 1B except for the following changes (see Table 1). Each word representation was a 300-dimensional vector, similar to previous experiments. The number of dimensions was selected to maintain a similar structure in the model, 300 dimensions to represent context information and another 300 to represent item information. However, this time the 300 dimensions that were composed of the first 100 dimensions for orthographic representations, the next 100 dimensions for phonological representations, and the final 100 dimensions for semantic representations. The optimal mix of dimensions among orthographic, phonological, and semantic representations is beyond the scope of the current study, but dimensions were kept equal to prevent giving unequal weight to any specific representation.

The same parameters were used for the simulations in Experiments 1A and 1B with the full model. For the full model, the parameters were similar to those used with the model that only included semantic representation, except the learning rate (*L*) was slightly lower and the recall threshold (*T*) was slightly increased: *L* = 0.25, *g* = 0.03, *d* = 0.3, *T* = 0.40, *s* = 0.

### Simulation results

In this section, we briefly present the results of our simulation using the full model across the same measures. Our goal was to evaluate whether the model, with its more comprehensive representation of each feature of every word in our lexicon could still capture the main aspects of memory performance for semantically related lists (Experiment 1A) and semantically unrelated lists (Experiment 1B).

#### Performance across experiments and serial positions

**Fig. 18:**
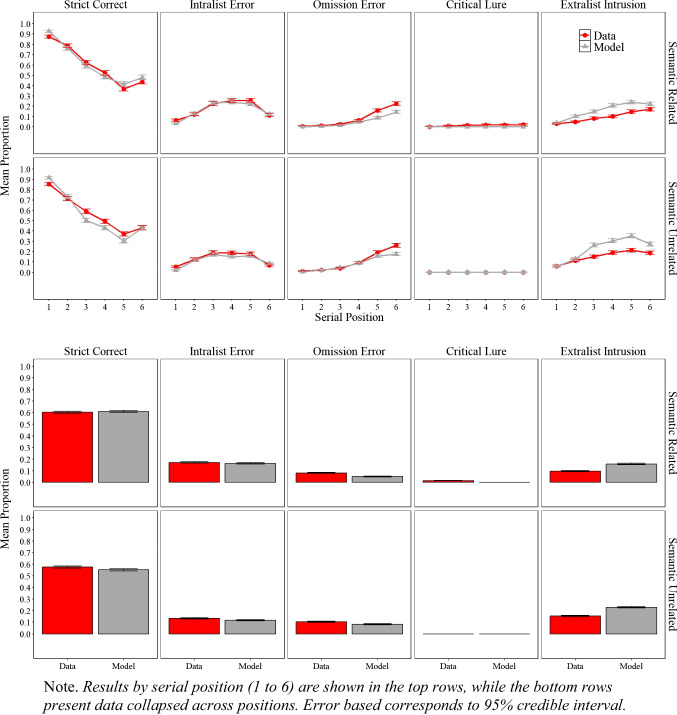
Full model (orthographic, phonological, and semantic representation embedded in the memory model) simulation results and experimental data for the mean proportion of correct recalls, intralist errors, omission errors, critical lure, and extralist error as a function of serial position in Experiment 1A (semantically related lists) and in Experiment 1B (semantically unrelated lists)

The results are presented in Fig. 18, alongside the experimental data for proportion correct, intralist error, omission, critical lure, and extralist error. Overall, despite changes in the lexicon, the model nearly tracked all main features including proportion correct, the standard serial position curve, intralist errors, omissions, and critical lures. The model also captured extralist errors with some minor discrepancies; it overpredicted the rate of extralist errors. Outside that discrepancy, the model provides a good fit to the overall data, and across serial positions for both related and unrelated semantic lists. More precisely, across Experiments 1A and 1B, the eCFM, utilizing orthographic, phonological, and semantic representations, demonstrated an excellent fit to the empirical data, achieving an ${R}^{2}$ = 0.96. Overall, the fit is comparable at the overall level to the model with only semantic representations.

#### Positional uncertainty

**Fig. 19:**
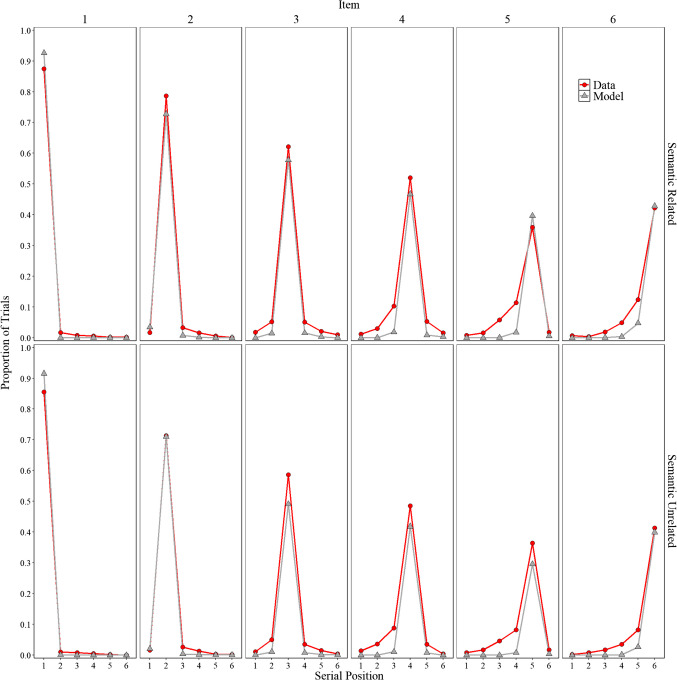
Full model (orthographic, phonological, and semantic representation embedded in the memory model) simulation results and experimental data for the proportion trials for each word (item 1–6) was recalled in each serial position (1–6) in Experiment 1A (semantically related lists) and in Experiment 1B (semantically unrelated lists)

For positional uncertainty presented in Fig. 19, as observed in Experiments 1A and 1B, the positional uncertainty curves of the experimental data and the model share many similarities, such as distributions that are locality-constrained (i.e., clustered) around the cued position, with more errors occurring at immediately adjacent positions than at positions farther away. Overall, the fit to the empirical data was good, ${R}^{2}$ = 0.98, and comparable to the model with only semantic representations. However, again the model is more constrained around adjacent positions than the experimental data, which shows a more gradual decrease for adjacent serial positions.

#### Critical lure and extralist errors

**Fig. 20:**
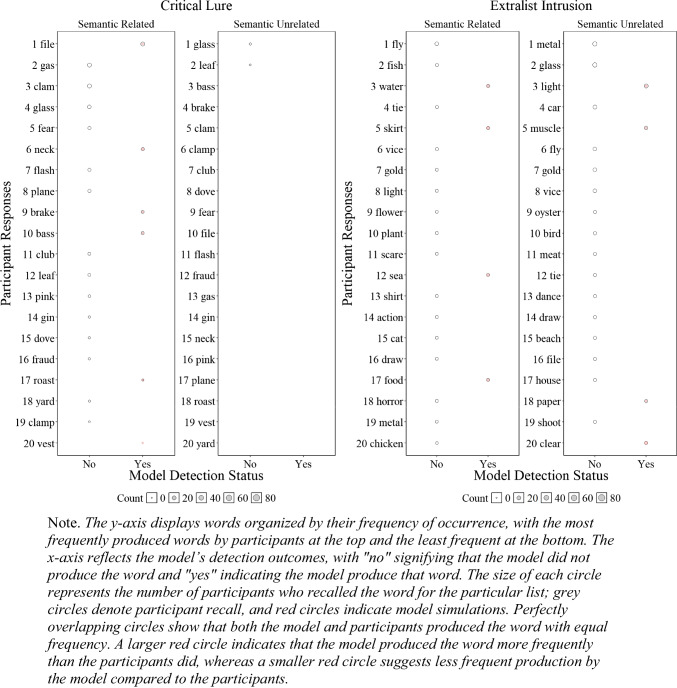
Illustration of the number of participants’ responses and number of model responses detections for the critical lure (**left panels**) and the 20 most common extralist intrusions collapsed across all lists (**right panels**) for Experiment 1A (semantically related words) and Experiment 1B (semantically unrelated words) with the full model (orthographic, phonological, and semantic representation embedded in the memory model)

In this section, we examine whether the full model can accurately track specific critical lures and the 20 most common extralist intrusions available in the model’s lexicon at the item level. Figure 20 illustrates how the model tracked these specific false memories and whether the frequency of these errors, represented by the size of the circles, aligns with those made by participants. For related lists, the model identified 5 out of 19 critical lures, while also producing 1 critical lure that was not identified by participants, compared to 11 out of 19 with the semantic-only representation. For semantic unrelated lists, participants produced 2 out of 20 critical lures, and the model did not identify any which is identical to the semantic-only model. For the top 20 most common extralist errors, the model identified 4 out of 20 for both related and unrelated lists, slightly outperforming the semantic-only model, which captured 1 and 3 out of 20 for the related and unrelated experiments, respectively. This suggests a slight improvement in capturing general extralist errors relative to critical lures with a more comprehensive lexicon.

#### Similarity between experimental data and model’s most common extralist errors

**Fig. 21:**
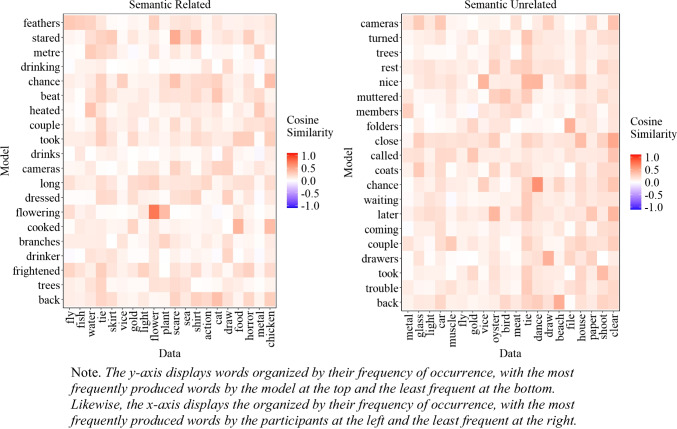
Cosine similarity matrix between the 20 most common extralist intrusions collapsed across all lists produced by the participants (x-axis) and the full model with orthographic, phonological, and semantic representation embedded in the memory model (y-axis) for Experiment 1A (semantically related lists) and Experiment 1B (semantically unrelated lists)

In this section, we examine the cosine similarity matrix between the 20 most common extralist errors made by participants and those generated by the model, aggregated across all lists, which is presented in Fig. 21. As shown in Fig. 21, the words produced by the model were related to those produced by participants, with some sharing orthographic and phonological similarities (e.g., 'dance' and 'chance') and others semantic without orthographic and phonological similarity (e.g., 'file' and 'folders'). Overall, there are some differences in the words produced by the most common extralist errors from the full model (100 dimensions for orthographic representations, the next 100 dimensions for phonological representations, and the final 100 dimensions for semantic representations) compared to those produced by the semantic-only representation model (300 dimensions semantic representations). However, these results support the value of embedding a lexicon to capture a more diverse array of extralist errors.

#### Word-level predictions

**Fig. 22:**
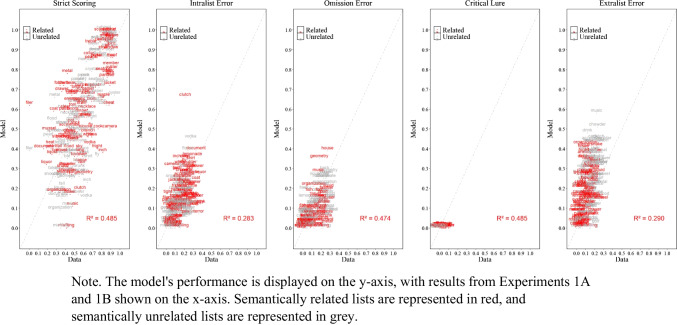
Simulation results of the full model (including orthographic, phonological, and semantic representations embedded in the memory model) and experimental data for the mean proportion of times each word was scored as strictly correct, intralist error, omission, critical lure, and extralist error, along with the overall fit for each scoring procedure in Experiment 1A (semantically related lists) and Experiment 1B (semantically unrelated lists)

In this final section, we leverage the comprehensive lexicon to make word-level predictions. Specifically, in Fig. 22, we examined the relationship between the model and data for related and unrelated materials to classify each word into the following categories: proportion correct (the likelihood of each word being recalled in its presented position), intralist error (the likelihood of each word being recalled in a different position), omission (the likelihood of each word not being recalled), critical lure (the likelihood of each word being replaced by a critical lure), and extralist error (the likelihood of each word being replaced by a word that was not studied and not a critical lure).

Overall, we observe that the model tracks not only the overall performance (Fig. 18) but also the word-level performance with a reasonable degree of accuracy, with fits ranging from ${R}^{2}$ = 0.28 to 0.49. It is evident that the model does not perfectly track the classification of all words; for example, it slightly overpredicts extralist errors for some words compared to the data. The novelty of this approach lies in its ability to determine if the processes accounting for average performance can also account for word-level predictions. For comparable investigations using lexicons corresponding to the study material in previous experiments, please refer to the **OSF** page associated with this manuscript.

### Discussion

In Experiments 1A and 1B, we captured many key features of memory performance with the eCFM by embedding a lexicon that contains semantic relationships among words. Here we address an important potential limitation by extending the representation to include orthographic, phonological, and semantic relationships among words. Despite these changes in representation, the model provides a good overall level and item level fit to the data, with similar issues in capturing the specificity of the positional uncertainty curves (e.g., more locality-constrained than the experimental data). The full model also slightly overpredicts extralist errors. However, at the item level for related lists, it performs slightly worse in capturing the specific critical lures but slightly better for both related and unrelated lists for the most common extralist intrusions. The results from these simulations provide initial success in capturing memory performance with a more comprehensive representation of each word.

## Phonological: Simulation full model

The results from our previous simulations, using the full model for the semantically related and unrelated experiments, provide strong evidence that a model incorporating orthographic, phonological, and semantic representations can still effectively capture memory performance. Before concluding the success of embedding a more comprehensive representation scheme into the model, we aimed to evaluate its efficacy for phonologically related lists (Experiment 2A) and phonologically unrelated lists (Experiment 2B). Thus, in the subsequent sections, we will examine whether this full model can accurately track the performance of phonologically related and unrelated materials.

### Method

#### Simulation parameters

The simulation details were similar to the semantic simulation with the full model (see Table 1) except we now simulated Experiment 2A (phonological related) and Experiment 2B (phonological unrelated). The parameters were identical to those used with the model containing only phonological representation except for the recall threshold (*T*) which was identical to the simulation with the full model of the experiments involving semantically related and unrelated materials: *L =* 0.215*, g =* 0.03*, d =* 0.3*, T =* 0.40*, s =* 0.

### Simulation results

In this section, we briefly present the main findings from our simulations using the full model for the phonologically related lists in Experiment 2A and the phonologically unrelated lists in Experiment 2B.

#### Performance across experiments and serial positions

**Fig. 23:**
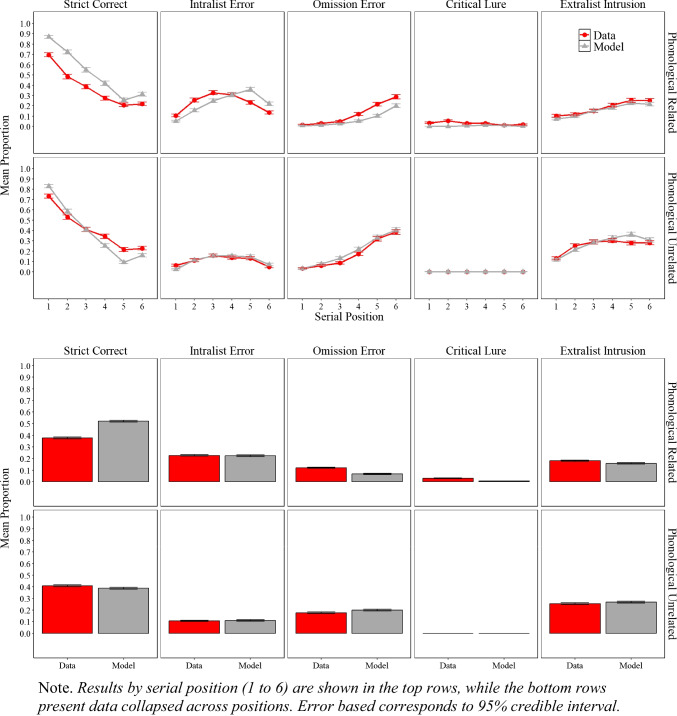
Full model (orthographic, phonological, and semantic representation embedded in the memory model) simulation results and experimental data for the mean proportion of correct recalls, intralist errors, omission errors, critical lure, and extralist error as a function of serial position in Experiment 2A (phonologically related lists) and in Experiment 2B (phonologically unrelated lists)

As shown in Fig. 23, similar to the model with phonological representations, the full representation model captures the pattern or proportion of correct responses. Despite minor discrepancies, it better captures memory for items presented early, the pattern of intralist errors, fewer intralist errors for early presented items, critical lures, and extralist errors, with more errors occurring in later positions. However, it slightly underpredicts the proportion of omissions for related lists at later serial position (e.g., 4, 5, 6). Nonetheless, despite these minor discrepancies, the eCFM—equipped with combined orthographic, phonological, and semantic representations—provides a reasonably good fit to the empirical data for both experiments, achieving slightly superior value than the model with phonological representation only with ${R}^{2}$= 0.91.

#### Positional uncertainty

**Fig. 24:**
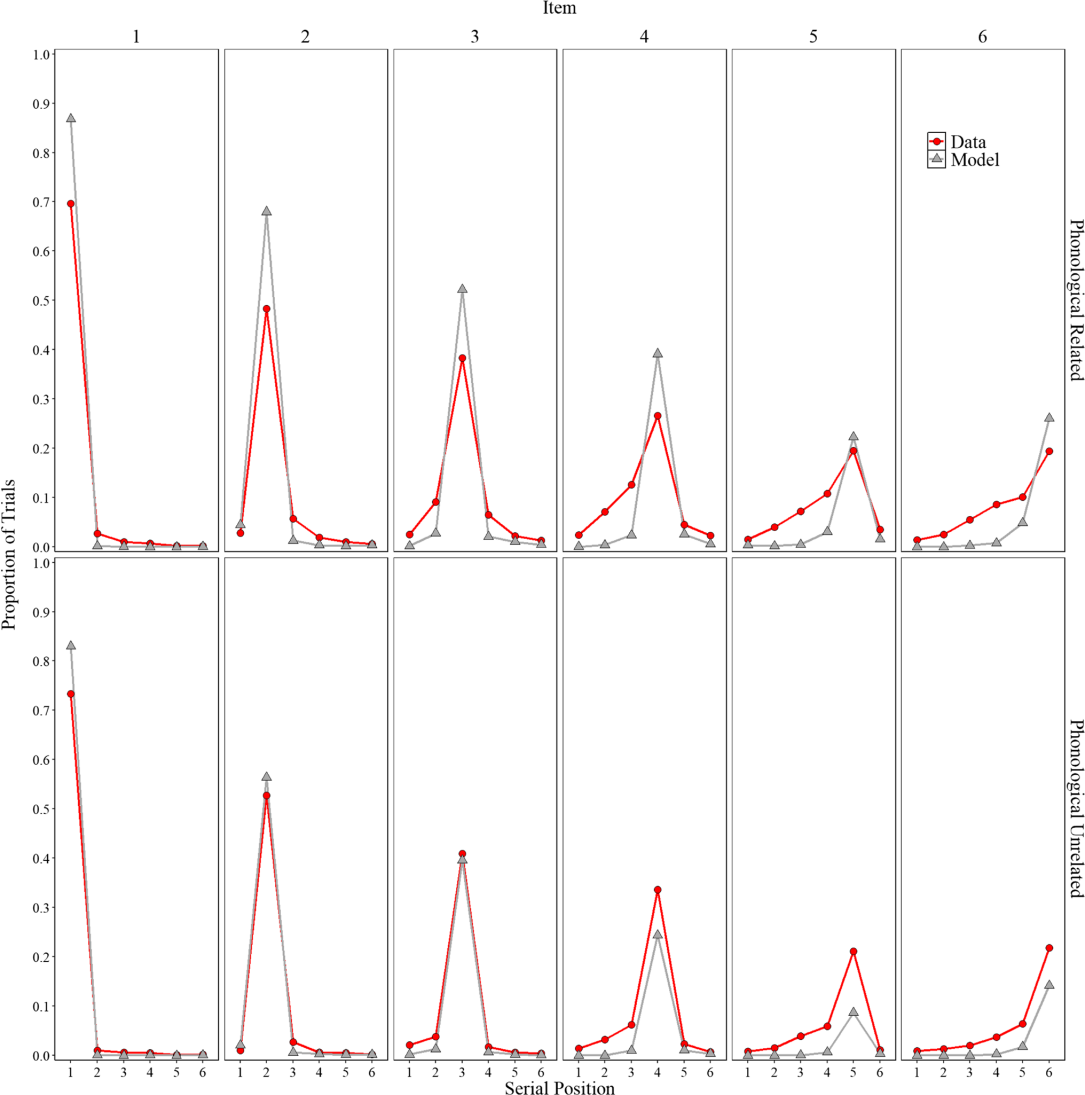
Full model (orthographic, phonological, and semantic representation embedded in the memory model) simulation results and experimental data for the proportion trials for each word (item 1–6) was recalled in each serial position (1–6) in Experiment 2A (phonologically related lists) and in Experiment 2B (phonologically unrelated lists)

Like the model with only phonological representations, the full model captures many key features for each position of the positional uncertainty (see Fig. 24), albeit with the distributions again locality-constrained around the presented position. However, similar to previous simulations, the model exhibits a slower decline in recall rates across adjacent positions, ${R}^{2}$= 0.94.

#### Critical lure and extralist errors

**Fig. 25:**
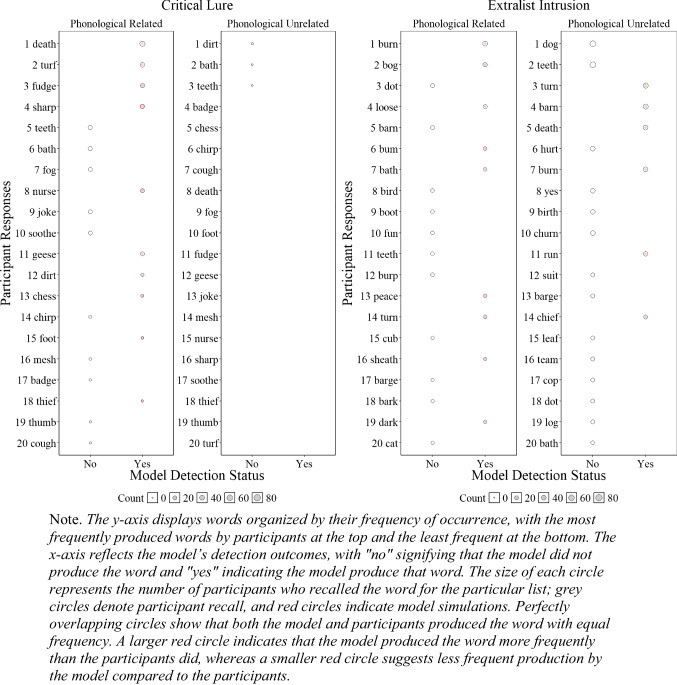
Illustration of the number of participants’ responses and number of model responses detections for the critical lure (left panels) and the 20 most common extralist intrusions collapsed across all lists (right panels) for Experiment 2A (phonologically related words) and Experiment 2B (phonologically unrelated words) with the full model (orthographic, phonological, and semantic representation embedded in the memory model).

In this section, we briefly assess whether the full model accurately tracked the specific critical lure and the 20 most common extralist intrusions available in the model’s lexicon. As shown in Fig. 25, the full model detected specific false memories with a degree of accuracy. For the phonologically related lists, the model identified 10 out of 20 critical lures, compared to 17 with the phonological-only representation model, with only minor discrepancies in frequency. For unrelated lists, like previous simulations, the model did not produce the participants 3 of 20 critical lures. In terms of extralist errors, for the related lists, the model captured 9 out of the 20 most common errors, compared to 5 with the phonological-only representation. For the unrelated lists, it captured 6 out of 20, compared to 11 with the phonological-only representation. Overall, while the full model lost some level of precision in capturing extralist errors for unrelated lists and critical lures for related lists, it gained precision in detecting extralist errors for phonologically related lists. We attribute the difference to participants’ encoding focus; if participants focused on phonology at study as the lists encouraged, including semantic and orthographic information serves to misrepresent that encoding focus.

#### Similarity between experimental data and model’s most common extralist errors

**Fig. 26:**
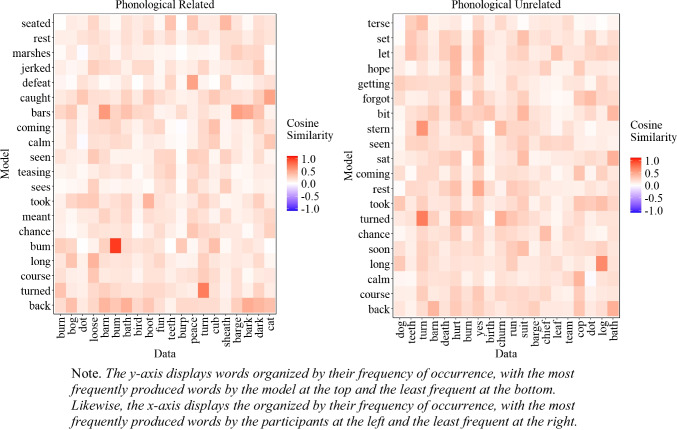
Cosine similarity matrix between the 20 most common extralist intrusions collapsed across all lists produced by the participants (x-axis) and the full model with orthographic, phonological, and semantic representation embedded in the memory model (y-axis) for Experiment 2A (phonologically related lists) and Experiment 2B (phonologically unrelated lists)

Once again, we examined the similarity between the most common extralist intrusions produced by the model and those produced by participants, this time using the full representational model. As illustrated in Fig. 26, the most common responses of the participants matched those of the phonological-only model less often than identical matches seen in Fig. 26. However, there is some level of similarity between the most common errors produced by the model and those made by participants. Overall, these simulations suggest that embedding orthographic, phonological, and semantic representations can capture specific aspects of memory errors. However, additional work may be required to accurately capture the specificity of human error, such as exploring the potential weighting in terms of representations, encoding strategies, and task characteristics.

#### Word-level predictions

**Fig. 27:**
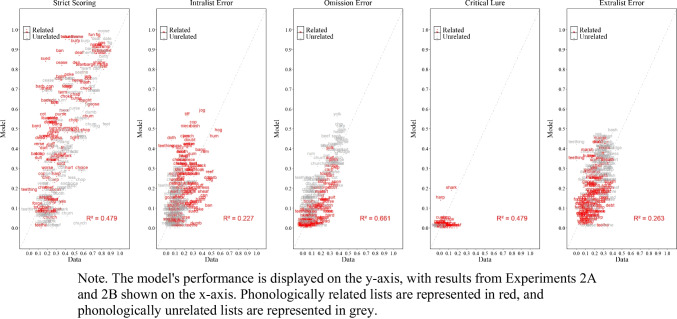
Simulation results of the full model (including orthographic, phonological, and semantic representations embedded in the memory model) and experimental data for the mean proportion of times each word was scored as strictly correct, intralist error, omission, critical lure, and extralist error, along with the overall fit for each scoring procedure in Experiment 2A (phonologically related lists) and Experiment 2B (phonologically unrelated lists)

As in the previous simulation for semantic materials, we analyzed the word-level predictions. More exactly, in Fig. 27, we explored the relationship between the model and the data for related and unrelated materials, classifying each word for proportion correct recall and for the different classes of error: intralist, omission, critical lure, and extralist.

Overall, Fig. 27 demonstrates that, similar to previous simulations, the model not only captures overall memory performance but also provides reasonable word-level predictions, with fits ranging from ${R}^{2}$ = 0.26 to 0.66. While the model is not yet perfect, this level of specificity is highly promising, indicating that simple assumptions can account for both macro and micro-level aspects in memory performance.

### Discussion

Our aim was to examine whether extending the representation to include orthographic, phonological, and semantic relationships among words could still capture many key features of memory performance with the eCFM for phonologically related (Experiment 2A) and phonologically unrelated lists (Experiment 2B). Overall, the model provides an adequate overall and word-level fit to the data, albeit with some loss in the precision of word-level predictions. Despite this slight loss of precision, the results from these simulations provide additional evidence that a comprehensive representation of each word can still capture memory performance.

## General discussion

When traditional computational memory models of serial recall attempt to account for verbal memory performance of related and unrelated materials (e.g., semantically, phonologically, orthographically related or unrelated words or non-words), most can predict the overall pattern of veridical and error responses (e.g., Henson, 1998; Maylor et al., 1999; Saint-Aubin et al., 2021, 2023). However, nearly all models of serial recall do not encode or recall specific words. Consequently, they fall short in making predictions that match such specific behaviours in experiments, because they fail to capture the intrinsic relationships formed by our linguistic experiences. This oversight misses the complexity and richness inherent in natural language (e.g., Johns & Jones, 2010), which in turn affects short-term memory performance (e.g., Guitard et al., 2018, 2019, 2025; Hulme et al., 1991, 2003; Majerus, 2019; Neath et al., 2022; Roodenrys et al., 2022; Oberauer et al., 2018).

In this study, our goal was to overcome that limitation and evaluate an extension of traditional memory models of serial recall by embedding a lexicon derived from distributional models of semantic memory (eCFM). Our aim was to capture the complexity and richness of interactions in natural language for semantic, phonological, and orthographic information, to coordinate those within a process model of episodic memory and evaluate if that amalgamation can capture memory performance that more directly matches human memory performance.

Across six experiments, we provided converging evidence that eCFM, by embedding a lexicon, not only captures typical features such as the proportion of correct responses, intralist errors, and omissions but also predicts false recalls, defined by specific critical lures or more general extralist errors. These predictions were consistent with participants’ behavior for semantically (Experiments 1A and 1B), phonologically (Experiments 2A and 2B), and orthographically (Experiments 3A and 3B) related and unrelated lists in serial recall.

This was accomplished using a subset lexicon corresponding to the studied material (e.g., semantic lists and semantic representations) and a more comprehensive lexicon including orthographic, phonological, and semantic representations against which information retrieved in the echo is compared for report. Overall, adding a lexicon capable of capturing the intrinsic relationships between studied and non-studied information supports modelling verbal memory performance at an improved level of specificity. This enhancement enables us to determine if the basic mechanisms we proposed still hold within a more holistic model of memory, with important theoretical ramifications for our understanding of memory.

In the next section, we briefly summarize the empirical and computational findings before discussing future directions and implications.

### Empirical summary

Here we summarize the key results related to extralist errors. Our empirical findings are clear and consistent with previous results in serial recall (e.g., Maylor et al., 1999; McCormack et al., 2000; Tehan, 2010). When participants studied lists of words that were related to a specific critical lure, semantically (Experiment 1A), phonologically (Experiment 2A), or orthographically (Experiment 3A), they were more likely to recall that specific critical lure compared to when participants studied unrelated lists (Experiment 1B, Experiment 2B, Experiment 3B). However, the pattern reverses for semantic and phonological extralist errors that are not the critical lure: participants were more likely to make extralist errors with unrelated lists (Experiment 1B and Experiment 2B) compared to related lists; an empirical fact that might be predicted in principle but not in particular with random word representations.

These results extend previous studies in serial recall across orthographic, phonological, and semantic information for both related and unrelated word lists. The findings suggest that lists related to specific critical words constrain which words will be falsely recalled. Traditionally, the study of extralist errors and false memory has used the DRM paradigm (Deese, 1959; Roediger & McDermott, 1995), where studied materials (e.g., table, sit, legs, wood, chair) are related to a specific critical lure (e.g., desk). However, here, in line with previous research (Maylor et al., 1999; McCormack et al., 2000), we demonstrated that moving beyond this traditional paradigm allows for a richer and more complex investigation of extralist errors.

Not only were these extralist errors more common than critical lures (i.e., the traditional measure of false memory), but they were also more diverse and harder to reconcile with traditional computational accounts of memory, which struggle to precisely predict the likelihood of recalling a specific word. In addition, the detailed analysis revealed that the most common extralist errors were words that were never presented in the experiment (extra-experiment errors), further demonstrating the necessity of embedding a comprehensive lexicon that captures both presented and unpresented information. Moving forward, including both traditional DRM procedures and unrelated lists has potential to provide a richer and more comprehensive empirical dataset, helping to build a deeper understanding of these important human memory errors.

### Computational summary

Our computational results are straightforward to summarize and align well with the growing efforts to build comprehensive models that integrate advances in the study of knowledge, memory, and cognition (e.g., Chubala et al., 2016; Johns et al., 2012; Kimball et al., 2007; Mewhort et al., 2018; Monaco et al., 2007; Morton & Polyn, 2016; Osth et al., 2020; Osth & Zhang, 2023; Polyn et al., 2009; Reid & Jamieson, 2022, 2023; Steyvers, 2000).

In this study, we used eCFM as proof of principle for the value of integrating structured word representations into a memory model (Guitard et al., 2025). We extended the model by embedding semantic, phonological, and orthographic representations using a DSM, and for the first time within this framework, investigated its ability to track veridical memory performance (proportion correct, intralist errors, omissions) and extralist errors simultaneously at both the overall and item levels.

Across the experiments, we demonstrated that the model can track key features of memory performance across veridical and extralist errors for related and unrelated semantic, phonological, and orthographic studied materials such as the serial position function, the distribution of errors, and position uncertainty with some level of success. Our work also shows that the model can track false recall of specific critical lures as well as extralist words that were related to the word list but were not a “critical lure.” While the model is not complete and further work is needed (which we will briefly discuss in the future directions), we believe that integrating a lexicon of word representations into an episodic memory model of storage and retrieval illustrates a necessary next step to advance our investigations of human memory. Specifically, our model may not be perfect and there is likely a better solution, but it demonstrates a valuable framework leveraging existing theories of semantic and episodic memory that can be extended to explore the important interactions between knowledge and memory that are necessary to a full account of memory. It also offers a framework for making word specific rather than general predictions about people’s behavior in studies of serial recall.

## Implications and future directions

The implications of our results suggest that integrating structured representations for words provides valuable insights and predictive precision in our predictions of human memory. While there are potentially other solutions, we have provided clear evidence of the value of considering how information is represented and the implications for predicting recall at the word-level. Traditional models of serial recall have offered valuable theoretical insights that we leveraged in our framework. The solution we have implemented can likely be incorporated into existing models of memory (e.g., Brown et al., 2000; Brown et al., 2007; Burgess & Hitch, 1999; Henson, 1998; Nairne, 1990; Murdock, 1995; Saint-Aubin et al., 2021). This approach has been extremely fruitful in recognition (e.g., Johns et al., 2012, 2020; Osth et al., 2020) and free recall (Kimball et al., 2007; Sirotin et al., 2005) but remains relatively uncommon in accounting for serial recall (e.g., Guitard et al., 2025; Mewhort et al., 2018). We encourage researchers to integrate representations based on articulated theoretical frameworks to investigate whether the mechanisms implemented in general can be extended to provide an account of serial recall in the specific.

Although our framework provides good evidence for accounting for memory performance at the specific list level, it remains relatively simple. There are important future directions that we aim to investigate beyond the scope of this paper.

### Trial unit model of memory

Like many serial recall theories, our current implementation of the eCFM (e.g., Brown et al., 2007; Henson, 1998; Nairne, 1990) operates as a trial unit model. This means that information from previous trials does not affect current memory performance. However, as highlighted in our detailed analysis of extralist errors, this current version of the model falls short in accounting for several important findings, such as the influence of prior list intrusions—where participants recall information from earlier trials (e.g., Henson, 1998; Osth & Dennis, 2015)—and the effects of proactive interference, where earlier lists influence memory for the current list (e.g., Carroll et al., 2010; Beaudry et al., 2014; Ralph et al., 2011).

In our exploratory analysis, we found that participants made more prior list intrusions than subsequent list intrusions (i.e., recalling words from previous trials rather than words presented later in the experiment; a kind of control condition by comparison). However, the model produced a similar number of errors in both cases. This outcome was expected, as the model was not designed to address these factors, and previous trials were effectively removed from memory.

To address this limitation, we are working on extending the model to capture the more dynamic and continuous nature of memory. There are several potential approaches to achieving this, such as reducing the forgetting rate of previous trials (instead of completely forgetting earlier trials, slightly reducing their memory influence), or adding a list context similar to serial position effects but tailored to each list. This would allow items from the current trial to be more readily retrieved, while still accounting for the influence of previous trials. Regardless of the specific approach, both solutions aim to make current information more active than prior information without fully erasing the memory of previous trials. In our ongoing work, we will explore and evaluate these approaches to enhance the model and provide a better account of the continuous nature of human memory.

Importantly, the solution we propose—embedding a lexicon—is not incompatible with a continuous memory model. For example, Mewhort et al. (2018) used a large lexicon of 39,076 words represented by BEAGLE vectors in a holographic recall model, demonstrating how a lexicon can provide precise predictions regarding the release of proactive interference. This approach could be integrated into our framework to deepen our understanding of human memory and related phenomena, offering a more comprehensive model of memory. Thus, embedding a lexicon does not conflict with the inclusion of prior information in memory models. However, the challenge remains to develop a continuous memory model that incorporates the influence of prior trials in the current context (see, for example, Kimball et al., 2007; Sirotin et al., 2005, in the context of free recall).

### Representations

Currently, we have embedded orthographic, phonological, and semantic information to represent item information. We have not evaluated the optimal weighting between these parameters (see Reid et al., 2023a, b for possible solution in recognition). Based on our simulations with subset representations (e.g., semantic representations for semantic materials) and a comprehensive lexicon (orthographic, phonological, and semantic), it seems that participants attend more heavily to features that advance performance in the local task (e.g., focusing more on phonological features when studying phonologically-related materials). These tentative conclusions appear consistent with the notion that we attend to a subset of features based on task demand and re-attend to these specific features during the memory test (e.g., Caplan, 2023; Caplan & Guitard, 2024a, b). However, further work is needed to understand this dynamic at both encoding and retrieval before further implementation. In future work, we will systematically investigate these mechanisms and the optimal weightings between representations using Nosofsky’s work as a guideline for integrating attention weighting in the Generalized Context Model (GCM).

We have adopted a standard distributional model of semantics for our representations. It is likely that other representation structures would provide a richer understanding and integration of information. For example, we could integrate neurosemantic representations using neuroimaging techniques to extract representations of meaning from brain activity (e.g., Mitchell et al., 2008; Mason & Just, 2020; Just et al., 2010) or combine them to provide a richer understanding (see also Fyshe et al., 2014). We also assume that our representations are stable across participants, but it is clear that, although there are shared representations as revealed by the field of neurosemantics, our representations are also shaped by variations in a person’s language environment (e.g., Aujla, 2021; Jamieson et al., 2018; Johns, 2024; Montag et al., 2015; Vong et al., 2024). Future work will attempt to make sense of people’s performance more accurately by integrating and comparing different kinds of word representation schemes, perhaps using experiential optimization (Johns et al., 2018).

### Beyond a model of tasks

Our work has shown that the eCFM can be applied to serial recall, serial reconstruction of order (Guitard et al., 2025), and across studied materials, including words and non-words. Because it is based on MINERVA 2 (Hintzman, 1986), the framework can be extended to recognition (Reid et al., 2023a, b), cued recall, categorization, associative learning, decision making, and more (see Jamieson et al., 2022 for a review). Our next objective is to demonstrate that the model can capture key memory performance metrics within a comparable empirical and computational framework. Models of memory should seek to integrate data over a range of tasks and contexts. However, the field has largely focused on building models for specific tasks while giving limited attention to the general processes or principle of how memory works and how to integrate them (but see Surprenant, & Neath, 2009; Kahana et al., 2024). Our framework is neither unique nor novel; it uses simple processes that have been around for decades and demonstrates how they can be integrated to provide precise predictions of human memory performance. The framework is flexible and imperfect, but with additional work, it will demonstrate how simple assumptions grounded in previous effort and hard-earned wisdom can account for a broad range of findings from the study of human memory and cognition.

### Simon’s (1969) Parable of the ant

Independent of the specifics, our approach is informed by insights that Herbert Simon (1956) and others (Todd & Gigerenzer, 2012) have argued. Simon (1956) presented the argument that models of cognition often ignore the environment for the internal world. In doing so, our theories too often misattribute sophistication to complex processing mechanisms in the brain. Todd and Gigerenzer’s (2012) book provides a range of scenarios in which the case plays out. In both cases, the theorists have argued that a full account of memory might be better envisaged by assuming memory is a relatively simple process (as in MINERVA 2) but that it exhibits complex behaviour when operating against a structured environment (as in the representations from LSA). Our modelling in this paper bears the point out. By equipping a MINERVA 2 with a semantic memory to borrow representations from, we are able to demonstrate some sophistication of the memory system with regards to false recall and remembering of unstudied but reasonable words in error. However, the point is larger than that context alone and our account represents a branch of the ecological cognition school in the present and of Simon’s arguments about cognition from the past. In that sense, we not only see our work as joining current efforts to model memory performance at word-level precision but also a more general demonstration of old ideas on the importance of modelling representation and how those representations play out in remembering. Although we do not draw out those connections here due to an already lengthy paper, we plan to draw those connections more explicitly in future work.

## Conclusion

It is well-established that verbal memory is fundamentally imperfect and reconstructive. However, traditional models have omitted a critical component: a lexicon that reflects how linguistic information is related. In this manuscript, we have demonstrated how the eCFM, by integrating structured representations that account for the intrinsic lexical relationships of verbal information, can overcome this limitation. Specifically, we have shown how the eCFM can account for veridical and false memory at both the macro (overall performance) and micro levels (word-level performance) across semantic, phonological, and orthographically related and unrelated materials. This work nicely extends the efforts of our predecessors in recognition (e.g., Johns et al., 2012, 2020; Osth et al., 2020) and recall (e.g., Kimball et al., 2007; Mewhort et al., 2018; Sirotin et al., 2005), demonstrating the value of having a more holistic model of memory. We encourage researchers to consider integrating memory within a more comprehensive architecture that coordinates accounts of semantic and episodic memory to move away from abstract predictions, towards more specific and testable predictions of human memory performance as a function of the specific words presented in study lists.

## Data Availability

All data and materials are available on the OSF page associated with the manuscript: https://osf.io/hmntw/. The stimuli are presented in the appendix.

## Appendix A

**Table 2:** Semantically related lists used in Experiment 1A and semantically unrelated lists used in Experiment 1B

Semantically Related Lists							
Item 1	Item 2	Item 3	Item 4	Item 5	Item 6	Lure	Mean Cosine Similarity
guitar	treble	drum	fish	music	boom	bass	0.404
stop	pedal	car	clutch	accelerate	speed	brake	0.481
oyster	seafood	shell	chowder	pearl	mussel	clam	0.322
hold	tight	vise	chisel	tool	metal	clamp	0.522
golf	member	ball	dance	organization	house	club	0.283
bird	peace	white	beak	bar	feather	dove	0.347
scared	fright	terror	anxiety	monster	snake	fear	0.389
tonic	alcohol	vodka	drink	liquor	drunk	gin	0.639
panther	pretty	purple	lemonade	rose	dress	pink	0.392
jet	air	fly	sky	travel	geometry	plane	0.360
cabinet	paper	folder	drawer	document	misfiling	file	0.475
light	camera	bulb	bright	back	flood	flash	0.341
fake	cheat	lie	crime	false	money	fraud	0.303
methane	station	energy	stove	heat	liquid	gas	0.315
window	crystal	cup	bottle	clear	jar	glass	0.387
tree	maple	branch	flower	fall	pot	leaf	0.360
throat	tie	collar	necklace	shoulder	long	neck	0.424
beef	pork	cook	turkey	oven	dinner	roast	0.498
suit	jacket	shirt	blouse	coat	pants	vest	0.642
mile	meter	inch	grass	stick	foot	yard	0.355
Semantically Unrelated Lists							
Item 1	Item 2	Item 3	Item 4	Item 5	Item 6	Lure	Mean Cosine Similarity
mile	jacket	cook	necklace	fall	jar	bass	0.241
guitar	meter	shirt	turkey	shoulder	pot	brake	0.219
stop	treble	inch	blouse	oven	long	clam	0.234
oyster	pedal	drum	grass	coat	dinner	clamp	0.224
hold	seafood	car	fish	stick	pants	club	0.270
golf	tight	shell	clutch	music	foot	dove	0.253
bird	member	vise	chowder	accelerate	boom	fear	0.168
scared	peace	ball	chisel	pearl	speed	gin	0.187
tonic	fright	white	dance	tool	mussel	pink	0.222
panther	alcohol	terror	beak	organization	metal	plane	0.193
jet	pretty	vodka	anxiety	bar	house	file	0.191
cabinet	air	purple	drink	monster	feather	flash	0.234
light	paper	fly	lemonade	liquor	snake	fraud	0.183
fake	camera	folder	sky	rose	drunk	gas	0.198
methane	cheat	bulb	drawer	travel	dress	glass	0.188
window	station	lie	bright	document	geometry	leaf	0.232
tree	crystal	energy	crime	back	misfiling	neck	0.205
throat	maple	cup	stove	false	flood	roast	0.284
beef	tie	branch	bottle	heat	money	vest	0.226
suit	pork	collar	flower	clear	liquid	yard	0.262

*Note*. Each row corresponds to a list and each column corresponds to the position of the item within the list. All participants were tested on all lists in a random order. The mean cosine similarity reflects the average similarity between studied words, including the critical lure

## Appendix B

**Table 3:** Phonologically related lists used in Experiment 2A and phonologically unrelated lists used in Experiment 2B

Phonologically Related Lists							
Item 1	Item 2	Item 3	Item 4	Item 5	Item 6	Lure	Mean Cosine Similarity
barge	ban	bang	bash	bat	batch	badge	0.382
chase	check	choice	guess	yes	less	chess	0.316
burp	chap	chop	churn	church	chip	chirp	0.365
date	dot	doubt	hurt	shirt	dart	dirt	0.255
fig	cog	dog	hog	jog	log	fog	0.353
fat	feet	fought	fight	fit	soot	foot	0.299
gas	goose	cease	piece	niece	lease	geese	0.419
seethe	sued	booth	soup	suit	soon	soothe	0.409
theme	sheaf	reef	leaf	beef	chief	thief	0.500
chum	dumb	hum	rum	sum	mum	thumb	0.397
birth	balm	barb	barn	bard	path	bath	0.327
calf	cuff	cob	con	cop	cot	cough	0.368
deaf	den	deck	debt	dead	doth	death	0.433
fun	fuzz	budge	judge	forge	nudge	fudge	0.460
jerk	choke	poke	soak	woke	yolk	joke	0.377
mush	mash	marsh	mess	met	men	mesh	0.368
noose	nerve	curse	purse	verse	worse	nurse	0.310
ship	shape	shop	shark	harp	carp	sharp	0.395
teach	team	tease	teethe1	heath	wreath	teeth	0.359
terse	term	turn	tiff	tough	surf	turf	0.436
Phonologically Unrelated Lists							
Item 1	Item 2	Item 3	Item 4	Item 5	Item 6	Lure	Mean Cosine Similarity
terse	team	shop	purse	met	yolk	badge	0.181
barge	term	tease	shark	verse	men	chess	0.143
chase	ban	turn	teethe	harp	worse	chirp	0.157
burp	check	bang	tiff	heath	carp	dirt	0.164
date	chap	choice	bash	tough	wreath	fog	0.146
fig	dot	chop	guess	bat	surf	foot	0.168
fat	cog	doubt	churn	yes	batch	geese	0.135
gas	feet	dog	hurt	church	less	soothe	0.147
seethe	goose	fought	hog	shirt	chip	thief	0.155
theme	sued	cease	fight	jog	dart	thumb	0.173
chum	sheaf	booth	piece	fit	log	bath	0.159
birth	dumb	reef	soup	niece	soot	cough	0.191
calf	balm	hum	leaf	suit	lease	death	0.190
deaf	cuff	barb	rum	beef	soon	fudge	0.205
fun	den	cob	barn	sum	chief	joke	0.169
jerk	fuzz	deck	con	bard	mum	mesh	0.135
mush	choke	budge	debt	cop	path	nurse	0.149
noose	mash	poke	judge	dead	cot	sharp	0.147
ship	nerve	marsh	soak	forge	doth	teeth	0.139
teach	shape	curse	mess	woke	nudge	turf	0.178

*Note*. Each row corresponds to a list and each column corresponds to the position of the item within the list. All participants were tested on all lists in a random order. The mean cosine similarity reflects the average similarity between studied words, including the critical lure

## Appendix C

**Table 4:** Orthographically related three letters non-words lists used in Experiment 3A and orthographically unrelated three letters non-words lists Experiment 3B

Orthographically Related Lists							
Item 1	Item 2	Item 3	Item 4	Item 5	Item 6	Lure	Mean Cosine Similarity
tqs	txs	pcs	tcz	lcs	tjs	tcs	0.508
xdj	xdk	xlf	xnf	xdp	xtf	xdf	0.536
zqg	dkg	dng	dqb	sqg	fqg	dqg	0.506
qgv	wjv	wdv	wsv	wgp	bgv	wgv	0.507
dmr	qcr	qmp	fmr	qmg	qmh	qmr	0.495
bfk	bpg	wpk	spk	bph	bpd	bpk	0.498
psh	jxh	pxd	pxz	pnh	lxh	pxh	0.488
jbz	jpz	jwz	jkg	jkn	lkz	jkz	0.509
cfs	cvb	jfb	cfw	cfz	pfb	cfb	0.496
znp	qnw	zcw	znl	zsw	jnw	znw	0.493
vlt	rlc	rqt	plt	rlg	wlt	rlt	0.498
vsw	vgm	rsm	vsq	vtm	vlm	vsm	0.507
ztj	htn	hpj	htg	htk	hsj	htj	0.498
lcp	lqp	lwg	lwf	swp	ldp	lwp	0.508
szd	szl	skx	sbx	mzx	gzx	szx	0.490
gjn	kbn	kjw	kjf	kjs	kxn	kjn	0.506
frd	frz	brl	mrl	fnl	frk	frl	0.492
nbq	gbq	mbl	sbq	mcq	mxq	mbq	0.504
nht	lhc	ghc	nhd	qhc	nkc	nhc	0.493
gmd	gjd	gvz	gvl	bvd	wvd	gvd	0.490
Orthographically Unrelated Lists							
Item 1	Item 2	Item 3	Item 4	Item 5	Item 6	Lure	Mean Cosine Similarity
gmd	lhc	mbl	mrl	kjs	gzx	tcs	0.236
tqs	gjd	ghc	sbq	fnl	kxn	xdf	0.214
xdj	txs	gvz	nhd	mcq	frk	dqg	0.201
zqg	xdk	pcs	gvl	qhc	mxq	wgv	0.229
qgv	dkg	xlf	tcz	bvd	nkc	qmr	0.199
dmr	wjv	dng	xnf	lcs	wvd	bpk	0.204
bfk	qcr	wdv	dqb	xdp	tjs	pxh	0.205
psh	bpg	qmp	wsv	sqg	xtf	jkz	0.219
jbz	jxh	wpk	fmr	wgp	fqg	cfb	0.231
cfs	jpz	pxd	spk	qmg	bgv	znw	0.199
znp	cvb	jwz	pxz	bph	qmh	rlt	0.216
vlt	qnw	jfb	jkg	pnh	bpd	vsm	0.194
vsw	rlc	zcw	cfw	jkn	lxh	htj	0.238
ztj	vgm	rqt	znl	cfz	lkz	lwp	0.237
lcp	htn	rsm	plt	zsw	pfb	szx	0.227
szd	lqp	hpj	vsq	rlg	jnw	kjn	0.223
gjn	szl	lwg	htg	vtm	wlt	frl	0.267
frd	kbn	skx	lwf	htk	vlm	mbq	0.206
nbq	frz	kjw	sbx	swp	hsj	nhc	0.223
nht	gbq	brl	kjf	mzx	ldp	gvd	0.179

*Note*. Each row corresponds to a list and each column corresponds to the position of the item within the list. All participants were tested on all lists in a random order. The mean cosine similarity reflects the average similarity between studied words, including the critical lure

## Appendix D

Illustration of the free parameters of the models. *L* denotes the base learning rate, *g* represents the rate at which encoding decreases with serial position, and *d* reflects the degree of similarity or dissimilarity between successive serial positions in memory for a study list. The top left panel shows the impact of *L*, the base learning rate, when *g* is set to 0 across all positions. The top right panel demonstrates the effect of *g* when *L* is fixed at 1. The four bottom panels display the cosine similarity of serial position representations as a function of *d*. These panels can be interpreted by cross-referencing the numbers within the graph with the serial positions on the x-axis. For example, the cosine similarity profile at serial position 3 in each graph, indicated by the green line with points labelled “3,” shows the similarity of each serial position from 1 through 6 to the representation of the third serial position

## Code availability

The code for simulations is available on the OSF page associated with the manuscript: https://osf.io/hmntw/.
